# A tumor microenvironment responsive biodegradable CaCO_3_/MnO_2_- based nanoplatform for the enhanced photodynamic therapy and improved PD-L1 immunotherapy

**DOI:** 10.7150/thno.37586

**Published:** 2019-09-21

**Authors:** Yanlei Liu, Yunxiang Pan, Wen Cao, Fangfang Xia, Bin Liu, Jiaqi Niu, Gabriel Alfranca, Xiyang Sun, Lijun Ma, Jesus Martinez de la Fuente, Jie Song, Jian Ni, Daxiang Cui

**Affiliations:** 1 Institute of Nano Biomedicine and Engineering, Shanghai Engineering Research Centre for Intelligent Diagnosis and Treatment Instrument, Department of Instrument Science and Engineering, School of Electronic Information and Electrical Engineering, Shanghai Jiao Tong University, 800 Dongchuan Road, Shanghai 200240, P.R. China; 2 National Center for Translational Medicine, Collaborative Innovational Center for System Biology, Shanghai Jiao Tong University, Shanghai 200240, P. R. China; 3 Tongren Hospital, Shanghai Jiao Tong University School of Medicine, 1111 XianXia Road, Shanghai, 200336, China.

**Keywords:** MnO2, Photodynamic therapy, PD-L1, Tumor immunotherapy

## Abstract

The low efficiency of photodynamic therapy (PDT) is caused by tumor hypoxia and the adaptive immune resistance/evasion of tumor cells, while the currently emerging immune checkpoint therapy restores the intrinsic immune capacities but can't directly attack the tumor cells.

**Methods:** Herein we report an integrated nanoplatform that combines PDT with immunotherapy to enhance photodynamic therapeutic effects and simultaneously inhibit tumor cells resistance/evasion. To achieve this, we fabricated Mn@CaCO_3_/ICG nanoparticles and loaded them with PD-L1-targeting siRNA.

**Results:** Thanks to the protection of CaCO_3_ on the loaded ICG and the oxygen produced by MnO_2_, an enhanced photodynamic therapeutic effect * in vitro* was observed. * In vivo* experiments demonstrated that the nanoplatform could efficiently deliver the loaded drug to the tumor tissues and significantly improve tumor hypoxia, which further contributes to the therapeutic effect of PDT * in vivo*. Moreover, the synergistic benefits derived from the siRNA, which silenced the checkpoint gene PD-L1 that mediates the immune resistance/evasion, resulted in a surprising therapeutic effect to rouse the immune system.

**Conclusions:** The combination treatment strategy has great potential to be developed as a new and robust method for enhanced PDT therapy with high efficiency and a powerful antitumor immune response based on PD-L1 blockade.

## Introduction

Photodynamic therapy (PDT) is based on the effect of a photo-sensitizer taken up by tumor cells which generates reactive oxygen species under the irradiation of a laser at a specific wavelength, leading to photoablation of the tumor cells and subsequent cell death 1-4. Due to their minimally invasive modalities, highly spatial and temporal controllability and specific lesion destruction, PDT holds a great promise to overcome the limitations of traditional cancer treatments 5, 6. In addition to killing tumor cells directly, PDT could simultaneously induce cytotoxic lymphocyte (CTL) mediated anti-tumor immunities and modulate the immunosuppressive microenvironment inside the tumor, thereby facilitating the killing of tumor cells 7, 8. However, the adaptive immune resistance/evasion of tumor cells strongly affects the efficiency of this immune response 9. Tumor cells resist the host immune response through the adaptive immune resistance/evasion in order to realize self-protection. , in which both programmed cell death receptor 1 (PD-1) and its ligand programmed death-ligand 1 (PD-L1) are key immune checkpoint molecules 10. Due to the high expression of PD-L1 in most of malignant tumor cells, the secretion of cytokines and the proliferation and activities of T cells are inhibited dramatically through the specific binding to PD-1 expressed on the surface of T cells, eventually leading to immune resistance/evasion 11.

Indocyanine green (ICG) have been approved by the Food and Drug Administration (FDA) for clinical use as an imaging and diagnostic agent^12^. ICG featuring notable near-infrared (NIR) optical properties within the optimal biological window of biomedical applications have been widely studied for NIR fluorescence-guided imaging 13, and the great potential in PDT and photothermal therapy (PTT) owing to deep permeation into tissues 14. However, the application of ICG is restricted by its intrinsic drawbacks, including poor photo-stability and thermal-stability in aqueous solution, and poor ^1^O_2_ production capacity, low quantum yield and rapid blood clearance 15. Previous studies have demonstrated that gold-nanoparticles could significantly increase the ^1^O_2_ production of photodynamic agents by localized electric field (LEF), and thus, the PDT efficacy is remarkably enhanced 16. However, these gold-nanoparticles also bring another challenge that they are extremely difficult for the organism to degrade or excrete 17, resulting in potential toxic effect.

In recent years, MnO_2_ and CaCO_3_ based nanomaterials are attracting more and more attentions for their ability to serve as carriers for specific delivery of loaded drugs, and as contrast agents for MR imaging and CT imaging 18-20. But beyond that, the most interesting feature is their capacity to modulate the tumor-microenvironment (TME) inside solid tumors 21-23. Exactly speaking, MnO_2_ based nanomaterials are able to trigger the decomposition of H_2_O_2_ existing in the TME into H_2_O and O_2_, so as to improve tumor hypoxia 24, 25. Meanwhile, CaCO_3_ based nanomaterials readily dissolve into biological components Ca^2+^ and CO_2_ when contact to the acidic TME, and thus making it somewhat relieved by H^+^ consumption^26^, ^27^.

**Scheme 1:**
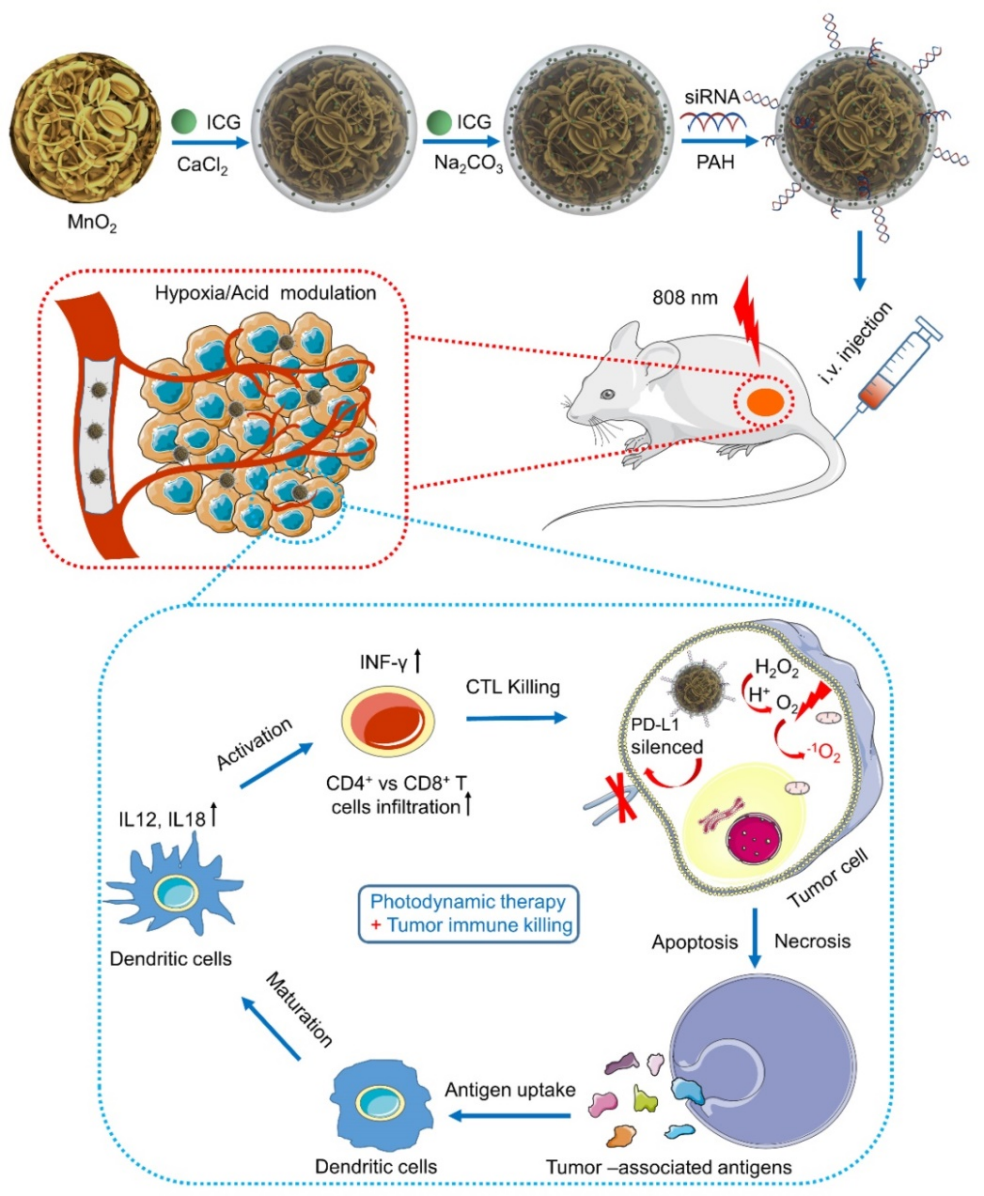
A schematic illustration of the synthetic route of Mn@CaCO_3_/ICG@ siRNA and the mechanism of the nanoprobe-mediated photodynamic tumor immunotherapy * in vivo*.

Herein, a nanoplatform of Mn@CaCO_3_/ICG@siRNA was designed and fabricated as shown in **Scheme 1.** In the nanoplaform, the walnut-like MnO_2_ nanoparticles were prepared through the reduction of potassium permanganate by PAH, and then the acquired MnO_2_ were modified with a pH-response cover layer of CaCO_3_ when ICG were simultaneously entrapped (Mn@CaCO_3_/ICG). PD-L1-targeting siRNA was loaded onto the positively charged Mn@CaCO_3_/ICG via electrostatic interaction to form the nanoplatform (Mn@CaCO_3_/ICG). In our work, the basic chemical and physical characteristics of the nanoplatform was detected. Their capability to modulate the TME and achieve the enhanced PDT * in vitro*/*in vivo* was evaluated. Furthermore, the anti-tumor immune responses induced by PD-L1 siRNA * in vivo* was demonstrated. The combination therapeutic effects of the enhanced PDT and anti-tumor immunities were finally investigated. Our study offers a powerful anti-tumor strategy exploiting comprehensive integration of PD-L1 siRNA with inorganic materials to exert their respective advantages, and highlights the enhanced tumor PDT and improved CTL-mediated anti-tumor immunities of the nanoplatform.

## Methods

### Materials

Potassium permanganate, polycyclic-aromatic hydrocarbons (PAH, 15000) were purchased from Sinopharm Chemical Reagent Co., Ltd. (Shanghai, China). Indocyanine green (ICG) was obtained from Sigma-Aldrich (St. Louis, USA). All cell culture products were obtained by GIBCO (Grand Island, NY, USA). Calcein- AM/PI double dyeing kit, annexin V-FITC/PI apoptosis detection kits were supplied by Yeasen Co. (Shanghai, China). Deionized (DI) water in 18.2 MΩ resistivity was applied in all aqueous solutions preparation.

PD-L1 siRNA labelled with FAM (siPD-L1) were purchased from Shanghai Gene Pharma Co.Ltd. (Shanghai, China), the sequences as follows: siPD-L1, sense: 5'-GGC GUU UAC UGC UGC AUA ATT-3'.

### Preparation of MnO_2_ nanoparticles

In our experiment, MnO_2_ nanoparticles were synthesized according to the one-step reduction method with some modification. In briefly, 20 μL of 23.6 mg mL^-1^ potassium permanganate solution was added to 12 mL of 8.43 μg mL^-1^ PAH aqueous solution, and then the mixture was ultrasound dispersed for 30 S. Following this step, the mixture was placed at room temperature without any shaking. After about 5 min the light purple reaction liquid turned light brown yellow, which indicated the formation of MnO_2_ nanoparticles. The as-prepared MnO_2_ nanoparticles were stored at 4 °C and used for the further experiment.

### Preparation of nanoplatform (Mn@CaCO_3_/ICG@siRNA)

50 mL of as-prepared MnO_2_ were purified following ultra-centrifuge process at 13000 rpm for 30 min and were washed twice with DI water, then gently re-suspended into 10 mL of DI water containing 2 mg of CaCl_2_ and 100 μg of ICG under moderate stirring for overnight, and then the over-dose chemicals were removed by another ultra-centrifugation at 13000 rpm for 30 min. Subsequently, the precipitate was gently added into 20 mL of DI water containing NaCO_3_ (50 μg mL^-1^) and ICG (5 μg mL^-1^), and the mixture was kept under moderate in the dark for 24 h. Finally, the resultant was washed twice times with DI water (13000 rpm, 30 min) to obtain the purified Mn@CaCO_3_/ICG.

In next step, the preparation of Mn@CaCO_3_/ICG@siRNA is based on the electrostatic interaction between Mn@CaCO_3_/ICG and siRNA. Briefly, 200 μL of PAH (23.6 mg mL^-1^) was added into 20 mL as-prepared Mn@CaCO_3_/ICG under mild stir for 6 h, then purified with DI water (13000 rpm, 30 min). Next, the sediment (The concentrations of Mn: 1-64 μg) was resuspended in 20 mL DI water containing siRNA (50 nmol), followed by shaking for 3 h. Finally, the Mn@CaCO3/ICG@siRNA were collected after the repeated centrifugation with DI water (11000 rpm, 30 min). Finally, the optimal binding ratio of the siRNA by polyacrylamide gel electrophoresis. All the components were reserved at 4 °C for further utilization.

### Characterization of nanoplatform

The morphology of nanoprobes was characterized by TEM, SEM and AFM, respectively. Elemental analysis was acquired with energy-dispersive X-ray from TEM (200 kV). X-ray photoelectron spectroscopy (XPS) was exerted to explore the chemical bonding configurations of MnO_2_ nanoparticles. Dynamic light scattering and zeta potential of these nanoprobes were measured using a NICOMP 380 ZLS Zeta Potential/Particle sizer. The UV/vis absorption spectra of ICG, MnO_2_ and Mn@CaCO_3_/ICG@siRNA were estimated respectively by using a Varian Cary 50 spectrophotometer. And the responding Fluorescence spectra were observed on a Hitachi F-4600.

### Profile of drug loading and release at different pH

The amount of loaded ICG in Mn@CaCO_3_/ICG@siRNA was measured using a UV-vis spectrophotometer at 785 nm. The ICG loading rate was determined by the following formula: Drug loading (%) = (Weight of drug loaded)/ (Weight of drug loaded and carrier) ×100%. The calculation of loaded siRNA in Mn@CaCO_3_/ICG@siRNA was achieved using the same method, with the exception that using different detection wavelength (525 nm) on a UV-vis spectrophotometer. In addition, the ICG and Ca^2+^ release experiment of Mn@CaCO_3_/ICG@siRNA was performed at different pH values (7.4, 6.5). As-prepared Mn@CaCO_3_/ICG@siRNA (1 mg) was dispersed in 1 mL of DI water at varying pH values. And then, the nanoprobes solution were transferred into a dialysis membrane (MWCO 5000) and immersed into 20 mL of the corresponding deionized water followed by continuous shaking in the speed of 100 rpm. Following this step, 1 mL of release media was separated for UV-vis evaluation and replenished with an same volume of DI water at the pre-set time points (0, 4, 8, 12, 16, 20, 24, 28 h). Finally, the content of released ICG and Ca^2+^ from Mn@CaCO_3_/ICG@siRNA was estimated using a Varian Cary 50 spectrophotometer.

### Singlet oxygen detection

Singlet oxygen sensor green (SOSG) was usually utilized for detecting the generation of ^1^O_2_, which was measured by monitoring the variation of recovered SOSG fluorescence density (excitation = 494 nm). In this experiment, the production of ^1^O_2_ from ICG and Mn@CaCO_3_/ICG@siRNA (with equal content of ICG) were determined by SOSG as sensitive probe in the concentration of 2.5×10^6^ M.

### Cells culture

Lewis cells were purchased from American Type Culture Collection (ATCC) and cultured in Dulbecco's modified Eagle's medium (DMEM) containing of 10% fetal bovine serum (FBS) with 5% CO_2_ at 37 °C in a humidified incubator. The expression of PD-1 receptor of the lewis cells were assessed in the presence of PE-PD-L1 using a FACS Calibur flow cytometry.

### Biocompatibility evaluation of nanoprobes

Lewis lung tumor cells were incubated at equally-distributed density of 1×10^4^ cells/well on 96-well plates. After 24 h incubation, with the replacement of fresh medium, containing a series concentration of PBS, ICG (0-32μg/mL), MnO_2_ (0-232μg/mL) and Mn@CaCO_3_/ICG@siRNA (Equivalent 0-32μg/mL) and further incubated 24 h, respectively. Finally, the cells viability was evaluated by the CCK-8 assay. In addition, the effect of nanoprobes on Lewis cells proliferation and viability were monitored using a Real-Time Cell Analyzer (RTCA Analyzer, Roche). Consistent with the pre-treatment, cells were plated in E-plates, which recorded the electronic impedance across microelectrodes and cells every 5 minutes during 72 h, finally the cell growth curves were recorded by RTCA Analyzer software.

### Cellular uptake of nanoprobes

Lewis lung cancer cells were seeded at a density of 5×10^4^ cells/well in 12-well plates. After 24 h incubation, replacing the medium with fresh medium dissolving PBS, ICG, siRNA and Mn@CaCO_3_/ICG@siRNA and cells were incubated in the dark for another 12 h. Cellular ICG and siRNA were observed by captured confocal fluorescence images to determine the cellular uptake and distribution in Lewis lung cancer cells using a Lecia SP8 STED 3X. Cell nuclei were stained blue with Hoechst 33342. Similarly, these cells were incubated with PBS, ICG, siRNA and Mn@CaCO_3_/ICG@siRNA in the dark for 6, 12 and 24 h, respectively, and then the fluorescence intensity of cellular ICG and siRNA were measured by a flow cytometry (BD Biosciences, Mountain View, CA). Besides, one part of the cells treated with Mn@CaCO_3_/ICG@siRNA was collected, following standard methods for TEM cell sample preparation. Finally, the distribution and degradation of nanoprobes in Lewis cell was observed by transmission electron microscopy.

### Western blot

The feasibility of Mn@CaCO_3_/ICG@siRNA to knock down PD-L1 in Lewis cells was evaluated using Western blot. The Lewis cells were incubated with PBS, Mn@CaCO_3_/ICG@siRNA for 48 h, and then The β-actin, PD-L1 were analyzed by Western Blot assay according to the standard protocols.

### Intracellular generation of O_2_

The intracellular generation of O_2_ was observed in the presence of [(Ru (dpp)_3_)]Cl_2_ using confocal fluorescence imaging. Because of the highly efficient quenching effect of O_2_on the fluorescence of this sensitive agent, [(Ru (dpp)_3_)]Cl_2_ is used as an O_2_-sensing probe. Lewis cells were incubated with 3 μM (Ru (dpp)_3_)]Cl_2_ in the dark for 4 h and further incubated with Mn@CaCO_3_/ICG@siRNA for different times (0, 1, 3, 9 h). After that, these lewis cells were observed using confocal fluorescence microscopy (excitation = 488 nm).

### Measurement of intracellular ROS generation

In our experimental, DCF-DA was selected to monitor the ROS generated by Mn@CaCO_3_/ICG@siRNA in Lewis cells. Lewis cells were seeded on 12-well and then incubated with PBS, ICG, MnO_2_ and Mn@CaCO_3_/ICG@siRNA. After 12 h culture, substituting with fresh medium containing DCF-DA (10 nM) for further incubation 20 min in the dark, followed by 808 nm laser irradiation (6 min, 0.5 W/cm^2^). After that, the Lewis cells were collected and analyzed by flow cytometry.

### * In vitro* NIR-laser mediated therapy

Lewis cells were cultured with PBS, ICG, MnO_2_ and Mn@CaCO_3_/ICG@siRNA (equivalent 16 μg/mL) for 12 h in the dark, respectively, and were washed three times with PBS to remove residuals in medium. After that, the Lewis cells were irradiated under an 808 nm NIR laser (0.5 W/cm^2^, 6 min). After laser irradiation, cells were cultured in fresh medium for another 6 h, and then one part of cells were identified by calcein AM/PI solution to observe the live and dead cells. And the others were harvested for following apoptosis analysis by flow cytometry.

### Delivery and Penetration capability of nanoprobes * in vitro*

Due to 3D Multicellular tumor spheroid (MCTS) can vividly mimic the tumor microenvironment of solid tumor. Therefore, a 3D MCTS with Lewis cells was used for assess the delivering and penetrating efficacy of nanoprobes. Briefly, lewis cells were seeded at a density of×10^4^ cells/well in a special 96-well (Nunclon Sphera). After 86 h incubation, ICG or Mn@CaCO_3_/ICG@siRNA was added into the medium for 12 h in the dark, and then confocal fluorescence microscope images of cellular ICG in the 3D MCTS were obtained using a confocal fluorescence microscope.

### Animals and tumor model

Female BALB/c nude mice (4 weeks of age) and male C57BL/6 mice (5 weeks of age) were purchased from Shanghai LAC Laboratory Animal Co. Ltd. Animals were housed in a SPF grade animal center during experiments process. All animal experiments were kept to the claim of Shanghai Jiao Tong University's institutional Animal Use and Care Committee. Lewis Lung cancer- model were established by subcutaneous injection of 5×10^6^ Lewis cells suspension in saline on the right hind limb.

### Tumor oxygenation measurements

A Vevo LAZR Photoacoustic Imaging System was used to imaging and measure tumor oxygen saturation (sO_2_) in situ in a series of time-points (0, 1, 3 and 8 h) after i.v. injection with Mn@CaCO_3_/ICG@siRNA (100 μg of Mn), and pre-injection time points were set as 0 h. The tumor oxygen saturation was measured at two excitation wavelengths (750 nm and 850 nm). All the photoacoustic images were performed with the same parameter settings.

### * In vivo* fluorescence, MR and CT imaging

In this experimental, A Bruker *In-Vivo* FPRO imaging system was utilized to make time-real observation of the distribution and metabolism of ICG in tumor-bearing mice. When the tumor size reached ≈ 100 mm^3^, Mice were injected by a lateral tail vein with Mn@CaCO_3_/ICG@siRNA (equivalent 10 mg kg^-1^), the NIR fluorescence images of ICG (exited by 710 nm; integrated in 30 s) were obtained at the pre-set time points(0, 0.5, 2, 4, 8). For T1-weight MR imaging, the tumor-bearing were intravenously injected with Mn@CaCO_3_/ICG@siRNA suspension at the dose of 10 mg Mn per mouse, MR imaging were implemented by a 1.5 T MR imaging instrument at a series of injection time points (0, 0.5, 1.5, 3, 6 h). For CT imaging, CT imaging were implemented by a CT imaging instrument at a series of injection time points (0, 0.5, 1.5, 3, 6 h). Here, the above-mentioned imaging modes acquired all images without changing the parameter settings. Blood samples (100 μL) were taken from the tumor-bearing mice at predetermined time points (0, 15, 60, 180, 540 minute) after the tail vein injection. And then, the Mn content in the blood was determined by an ICP-MS (Agilent 7500a).

### PDT and PD-L1 siRNA induced cytokine secretion and T cell proliferation

To evaluate the influence of PDT and PD-L1 on the proliferation, frequency and functions of CD4^+^ and CD8^+^ T cells, lewis bearing mouse were injected with the PBS, ICG, MnO_2_ and Mn@CaCO_3_/ICG@siRNA. Up to 8 hours after the i.v. injection, the tumor regions of injected-mouse were irradiated by a 808 nm laser for 6 min (1 min interval after 3 min) at a photo-density of 0.5 W/cm^-2^. The tumors were harvested at 5 days after laser-irradiation treatment, digested with collagenase IV, hyaluronidase and DNase for 30 min at 37 °C for preparation of single-cell suspensions. Before the surface staining, the suspensions were incubated with Fc Block for 15 min on ice, followed by surface staining with the various fluorescently labeled anti-mouse antibody. The pretreatment tumor samples were equally divided into two parts for DC cell staining and T cell staining. The specific T cells were stained with anti-CD3-APC (Biolegend, Clone: 17A2), anti-CD45-APC/Fire™ 750 (Biolegend, Clone: 30-F11), anti-CD8a-PerCP/Cyanine5.5 (Biolegend, Clone: 53-6.7), anti-CD4-FITC (Biolegend, Clone: RM4-5), FOXP3-PE (Bioscience, Clone: MF-14) antibodies according to the manufacturer's protocols. For analysis of mature of DC cells, the samples were stained with anti-CD45-APC/Fire™750 (Biolegend, Clone: 30-F11), anti-mouse I-A/I-E-PerCP/Cyanine5.5 (Biolegend, Clone: M5/114.15.2), anti-CD11c-APC (Bioscience, Clone: N418), anti-CD80-PE/Cy7 (Bioscience, Clone: 16-10A1), anti-CD86-PE (eBioscience, Clone: GL-1) antibodies. The mature of DCs were defined as the co-expression of CD80 and CD86. All samples washed there times with wash buffer before applying flow cytometric analysis. The secretion of pro-inflammatory cytokines including interferon-γ (IFN-γ), interleukin-12 (IL-12) and interleukin-18 (IL-18) were measured using ELISA kits at 5 days after each laser treatment.

### * In vivo* anti-tumor therapy

When tumor size reached 100 mm^3^, PBS, free ICG, MnO_2_ and Mn@CaCO_3_/ICG@siRNA was injected through a lateral tail vein. Up to 8 hours after i.v. injection, 808 nm laser was exerted to radiate the tumor site at a power of 0.5 W/cm^2^ with 1 min interval after every 3 min of the irradiate (irradiation twice). After the last irradiation treatment, tumor size was measured every four days by digital vernier caliper for 28 days. The mice were sacrificed and the tumors were collected 7 days after irradiation treatment, following standard methods for hematoxylin & eosin and TUNEL staining. Total proteins were collected from treatment and un-treatment tumor tissues, the β-actin and PD-L1 were analyzed by Western Blot assay according to the standard protocols.

### Statistical analysis

Data were presented as mean ± standard deviation, obtained from three independent parallel trials unless additional specified. Statistical significance was determined according to a two-tailed student′s t-test. A probability level of 95% (*P* < 0.05) was considered significantly different.

## Results and discussion

### Characterization of MnO_2_ and Mn@CaCO_3_/ICG@siRNA

**Figure 1:**
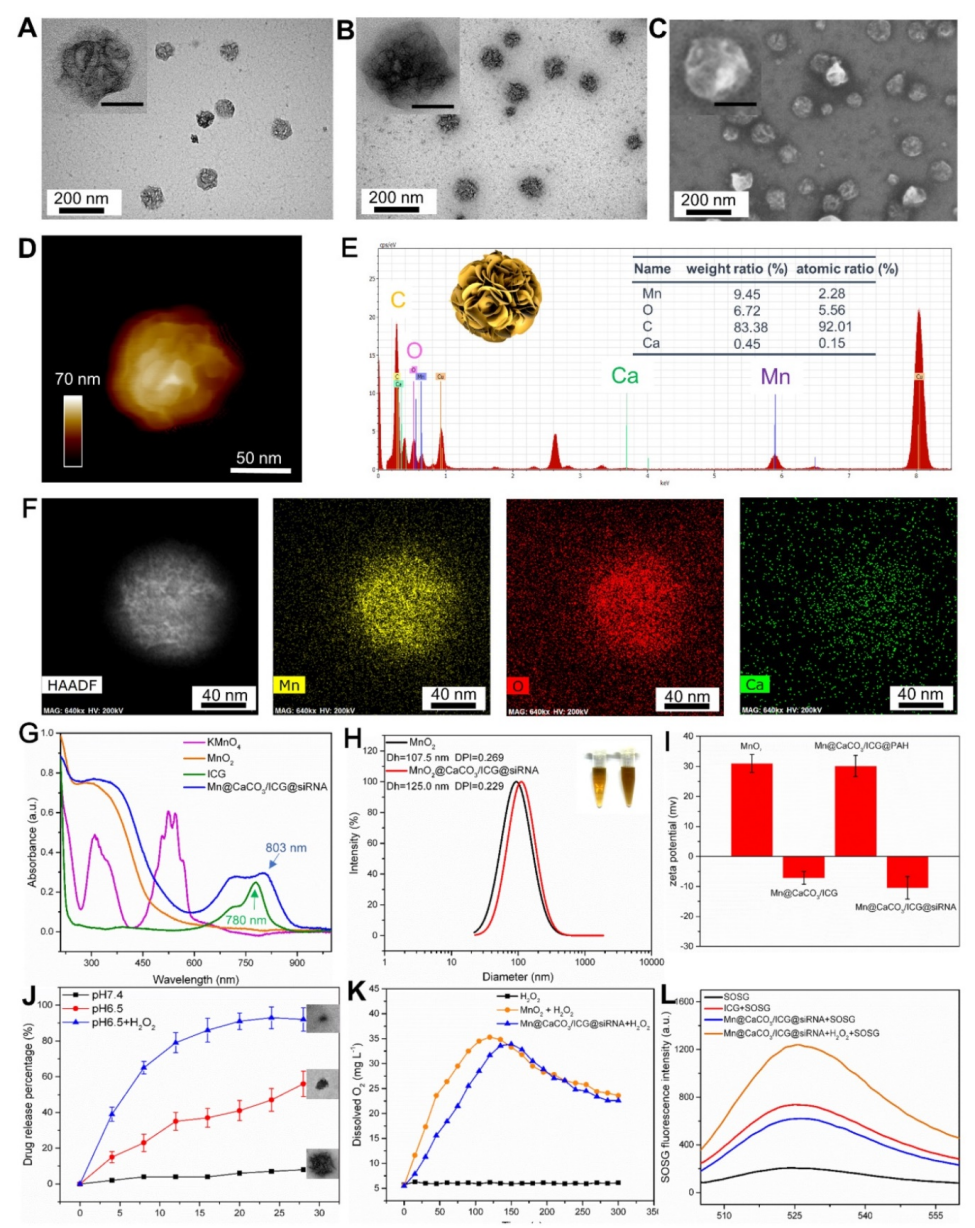
** Characterization of Mn@CaCO_3_/ICG@siRNA: (A)** TEM images of MnO_2_ nanoparticles. **(B)** TEM images of MnO_2_@CaCO_3_/ICG@siRNA. **(C)** SEM images of MnO_2_@CaCO_3_/ICG@siRNA. **(D)** AFM image of Mn@CaCO_3_/ICG@siRNA. **(E)** Energy dispersive X-ray spectroscopy of Mn@CaCO_3_/ICG@siRNA by STEM. All scale bars in the insets are 50 nm. **(F)** STEM images and the corresponding element mapping of Mn@CaCO_3_/ICG@siRNA: Mn; O; Ca. **(G)** UV-vis absorption spectra of KMnO_4_, MnO_2_ nanoparticles, free ICG and Mn@CaCO_3_/ICG@siRNA. **(H)** Hydrodynamic diameter of MnO_2_ and Mn@CaCO_3_/ICG@siRNA. The inset: digital images of MnO_2_ (a) and Mn@CaCO_3_/ICG@siRNA (b) in PBS. **(I)** Zeta potential of as-prepared MnO_2_, Mn@CaCO_3_/ICG, Mn@CaCO_3_/ICG@PAH and Mn@CaCO_3_/ICG@siRNA. **(J)** *In vitro* release profiles of the entrapped ICG from Mn@CaCO_3_/ICG@siRNA. **(K)** *In vitro* O_2_ production profiles of Mn@CaCO_3_/ICG@siRNA. **(L)** SOSG fluorescence spectra of Mn@CaCO_3_/ICG@siRNA.

The core of the nanoplatform (MnO_2_ nanoparticles) was initially synthesized according to a reduction method with some modifications 28. As expected, transmission electron microscopy images presented that the MnO_2_ nanoparticles established an prominent size uniformity and dispersibility in a form of a walnut-like shape (Figure 1A). Figure S1 presents the X-ray photoelectron spectroscopy (XPS) pattern of the as-prepared MnO_2_, which indicated the MnO_2_ particles have an oxidation state of +4 (two peaks, centered at 653.6 and 641.7 eV) 29. After covered by the layer of CaCO_3_ with simultaneously entrapped ICG (loading ratio: 11.5%), Mn@CaCO_3_/ICG was obtained. Finally, the siRNA-FAM were loaded on the surface of Mn@CaCO_3_/ICG by electrostatic adsorption, moment in which the nanoplatform (Mn@CaCO_3_/ICG@siRNA) is successfully fabricated (Figure 1 B, C). Simultaneously, the size and morphology of this nanoplatform was further characterized by AFM (Figure 1D). The observations of the core-shell structure were further validated by STEM imaging and EDS elemental mapping (Figure 1 E, F). As shown by Figure 1G, ICG has one main peak at 780 nm and a shoulder at 710 nm. After the CaCO_3_ layer entrapped with ICG on the surface of MnO_2_, the main peak of ICG exhibited a red-shift to 803 nm, suggesting that ICG is successfully loaded into the nanoprobes in aggregation state. The loading ratio of photosensitizer in Mn@CaCO_3_/ICG was 12.1 wt% by UV/vis spectra. ICG tends to form more stable aggregates at high concentrations in aqueous solution 30, thus declining the fluorescence of the monomers. As shown in Figure S2, the fluorescence quenching of loaded ICG was confirmed by fluorescence spectra of Mn@CaCO_3_/ICG, which further verifies that the loaded ICG is in a stable aggregation state. ICG molecules at higher concentration in water readily form aggregation state leading to a fluorescence decline. Moreover, we found that fluorescence quenching also occurred after siRNA was loaded onto the nanoplatform (Figure S3).

Based on this phenomenon we evaluated the optimal binding of siRNA to Mn@CaCO_3_/ICG by agarose gel electrophoresis assay (Figure S4), and it was finally determined that the optimal binding ratio of Mn@CaCO_3_/ICG:FAM-siRNA was 0.64 (expressed as 32 μg nanoplatform/50 nmol FAM-siRNA). The results of particles size analysis are shown in Figure 1H. MnO_2_ and Mn@CaCO_3_/ICG@siRNA have a narrow size distribution with average diameters of 107.5 ± 9.5 nm and 125 ± 10.5 nm, respectively. MnO_2_ presented a positive zeta potential of 31.0 ± 3.03 mV because the attached PAH has a mass of amide on the external. After entrapped by the CaCO_3_ layer with a great number of -OH groups, Mn@CaCO_3_/ICG showed a negative zeta potential of -7.12 ± 2.03 mV (Figure 1I). In order to effectively load siRNA by electrostatic adsorption, the surface of Mn@CaCO_3_/ICG is modified again with a second layer of PAH, which changes the zeta potential as 30.1 ± 3.03 mV, thereby helping to load the negatively charged siRNA on the Mn@CaCO_3_/ICG.

It is worth noting that the two inorganic materials (manganese dioxide and calcium carbonate), which constitute the nanoplatform, can be decomposed in acidic environments, according to previous studies 11, 26. Herein, the effects of acidic environment on the stability of nanoprobes were investigated. TEM images of Mn@CaCO_3_/ICG@siRNA after incubation in PBS at a variety of pH values for 12 h were recorded (as seen in Figure S5), the size and morphology of Mn@CaCO_3_/ICG@siRNA showed no significant change after being exposed to a pH value of 7.4, indicating that the nanoprobes were stable under the neutral conditions. In contrast, from TEM it can be clearly observed that the degree of degradation of the nanoprobes gradually increased with the decrease of pH. The nanoprobes exhibited acid-dependent degradation behavior due to the decomposition MnO_2_ and CaCO_3_ into Mn^2+^ ions and Ca^2+^ ions, respectively. In addition, O_2_ and CO_2_ were produced by this decomposition process, which may promote the release of the loaded drug and assist its diffusion into monomers.

We next investigated the pH-triggered release performance of the loaded drug from Mn@CaCO_3_/ICG@siRNA in varying pH (7.4, 6.5) environments. As expected (Figure 1J), the amount of ICG released from nanoprobes was below 10 % after 28 h incubation at a pH of 7.4, as CaCO_3_ and MnO_2_ were unable to be decomposed in this neutral environment. In contrast, a distinguished release of the loaded ICG from Mn@CaCO_3_/ICG@siRNA was activated when the pH value was decreased to 6.5. 16 hours after incubation in medium containing hydrogen peroxide with pH value of 5.0, the amount of ICG released reached up to 90 %. The release files of calcium ions further confirm the PH-responsive release characteristics of the nanoprobe (Figure S6). In addition, we investigated the stability of Mn@CaCO_3_/ICG@siRNA in various media (saline, PBS and medium containing 10 % fetal bovine serum). Next, we explored the stability of our nanoplatform in different media with a pH of 7.4. As shown in Figure S7, the nanoplatform was dispersed in an aqueous solution for 15 days and no color change as well as obvious aggregation occurred before and after dispersion. Besides, the diameter of the nanoplatform did not change during this period (Figure S8), indicating that the nanoplatform is extremely stable.

Previous studies have shown that MnO_2_ exhibited a high degree of reactivity and specificity toward H_2_O_2_ to produce O_2_ and Mn^2+^ by consuming H^+^ ions.^11^, ^31^ Therefore, we next investigated whether the trapped CaCO_3_/ICG would affect the reactivity of MnO_2_ toward H_2_O_2_. The nanoplatform was incubated with H_2_O_2_ in PBS at a pH value of 6.5 for 300 s, and the changes in O_2_ levels were continuously monitored during the process. As shown in Figure 1K, the O_2_ levels had no significant changes in H_2_O_2_ groups during the monitoring process. In contrast, the O_2_ produced by MnO_2_ and Mn@CaCO_3_/ICG@siRNA significantly increased during the first 100 s, then reached a peak of 35 and 33 mg/L respectively, and finally entered a plateau, suggesting that Mn@CaCO_3_/ICG@siRNA nanoparticles have almost the same capability to produce O_2_ as MnO_2_. Besides, it is notable that Mn@CaCO_3_/ICG@siRNA reached their maximum at 130 s, almost 30 s later than MnO_2_. We consider that CaCO_3_ on the surface of nanoprobes is responsible for this phenomenon, as MnO_2_ will not be exposed to hydrogen peroxide until CaCO_3_ is completely decomposed. In addition, we also explored the oxygen generation of the nanoplatform in different media. It can be clearly seen from Figure S9 that the nanoplatform generated a large amount of oxygen bubbles when H_2_O_2_ was present, and no oxygen bubbles were observed in the absence of it, indicating that the oxygen was produced by degradation of H_2_O_2_.

^1^O_2_ is a kind of highly reactive oxygen species considered to play a critical role in the efficacy of PDT 18. The production of ^1^O_2_ converted by ICG and Mn@CaCO_3_/ICG@siRNA was measured under irradiation using an 808 nm laser. The results are shown in Figure 1L. After 5 minutes of laser irradiation, the fluorescence intensity of Mn@CaCO_3_/ICG@siRNA was almost 2.6-folds greater than free ICG, indicating that Mn@CaCO_3_/ICG@siRNA has a stronger capacity to produce ^1^O_2_ under the same concentration of ICG. Combined with these experimental results above, we speculate that the enhanced ^1^O_2_ generation capacity is achieved through the following three ways: (Ⅰ) The CaCO_3_ layer enhances ICG stability thanks to the formation of loaded-ICG aggregates. (Ⅱ) The nanoprobe protects the loaded ICG from thermal/light induced degradation. (Ⅲ) the MnO_2_ core provides large amounts of enriched oxygen in local area to facilitate the generation of single oxygen.

### Cellular uptake

**Figure 2:**
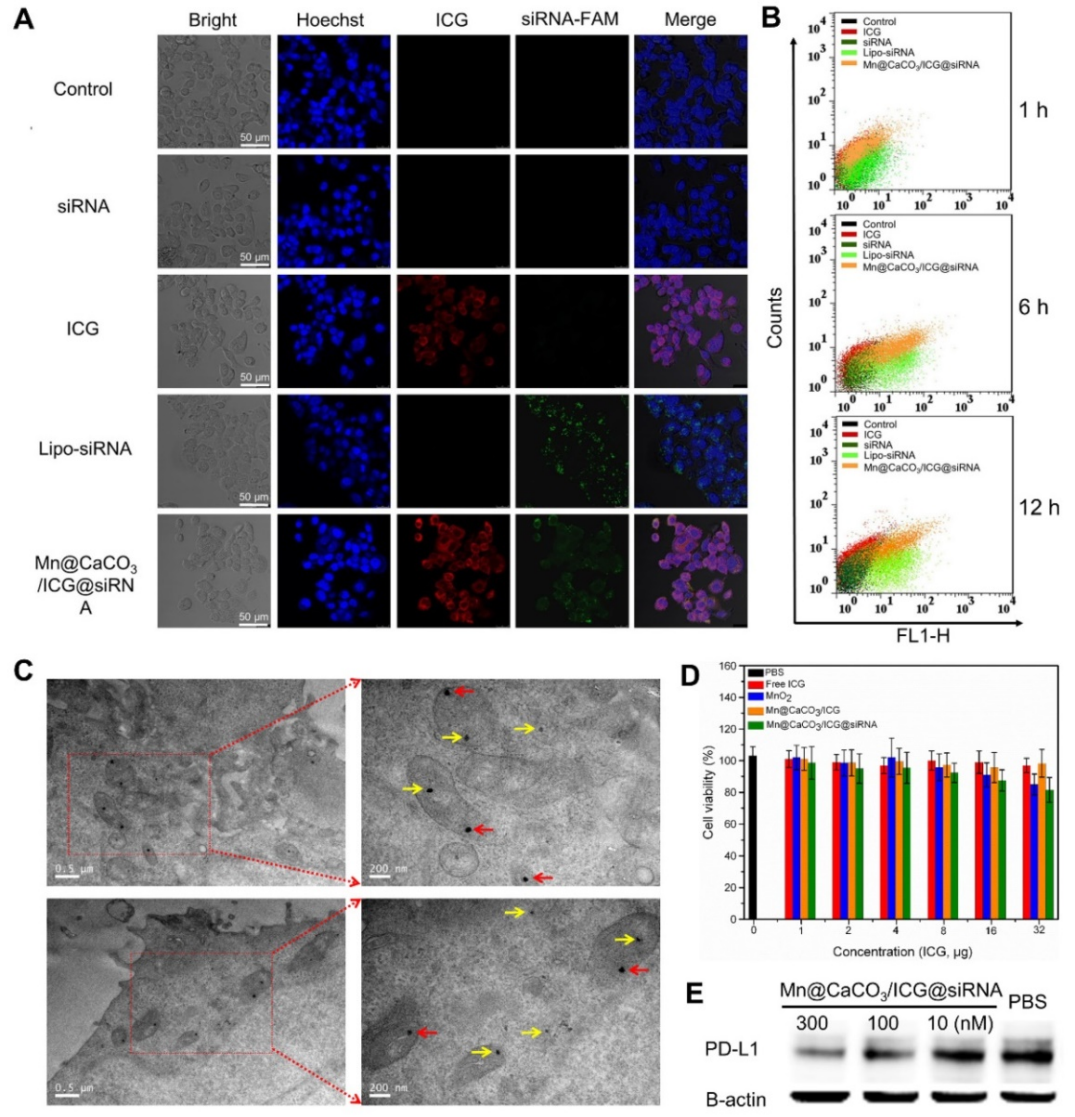
** (A**) Confocal fluorescence images of Lewis cells. **(B)** Flow cytometric analysis of intracellular fluorescence intensity of Lewis cells. **(C)** Representative TEM images of Lewis cells. The arrows in TEM images indicate the endocytosed nanoprobes. The yellow arrows and red arrows indicate the endocytosed nanoprobes with and without degradation, respectively. **(D)** The cell viability of LLC cells incubated with various concentrations of PBS, free ICG, MnO_2_, Mn@CaCO_3_/ICG and Mn@CaCO_3_/ICG@siRNA. **(E)** The PD-L1 protein expression in Lewis cells detected by Western blot.

Before performing the NIR-laser mediated therapy on Lewis lung carcinoma (LLC) cells, the cell uptake and intracellular distribution of Mn@CaCO_3_/ICG@siRNA were observed on account of their optical properties and pH-triggered degradation capability. Due to the slightly acidic character of endosomes/lysosomes and the cytoplasm, the loaded siRNA and entrapped photosensitizer were gradually released from the nanoplatform following the degradation of CaCO_3_ in acidic condition after endocytosis. Confocal images were taken to visualize Lewis cells incubated with PBS, ICG, siRNA, Lipo-siRNA and Mn@CaCO_3_/ICG@siRNA. As shown in Figure 2A, both free ICG and nanoplatform groups showed strong fluorescence signals from ICG. Whereas, those groups treated with the nanoplatform showed much stronger fluorescence, which demonstrated the prominent delivery and protection effects of the nanoplatform on ICG. Besides, similar results were obtained by observing the green fluorescence from siRNA. No obvious fluorescence signals were observed in the free siRNA groups, as siRNA cannot freely enter the cells without carriers and it is easily degraded outside the cell. However, for the Lipo-siRNA and nanoplatform incubation groups, the almost equal fluorescence signal can be observed from siRNA in the cytoplasm, indicating an effective delivery of siRNA by the nanoplatform into the cells. Moreover, red fluorescence from ICG and green fluorescence from siRNA were observed in those cells treated with Mn@CaCO_3_/ICG@siRNA, further suggesting an efficient cellular uptake of our nanoplatform. We then performed a quantitative analysis of the fluorescence intensity of cellular ICG and siRNA at different incubation time-points using flow cytometry. As shown in Figure 2B, the group treated with Mn@CaCO_3_/ICG@siRNA showed that the longer is the incubation period, the more loaded cargo is delivered into the tumor cells, which was not observed in other groups. These results fully proved that the nanoplatform is a promising nano-carrier for the delivery of particular cargo including ICG and siRNA to cancer cells, providing the prerequisite for successful PDT and silencing of genes.

To further explore the intracellular distribution and degradation of our nanoplatform in Lewis cells, TEM imaging was used intuitively to display the internalization of these nanoplatforms. TEM images are shown in Figure 2C, the nanoplatform was observed in the organelles and cytoplasm without a particular pattern of distribution. However, one obvious difference is that those nanoplatforms located inside the mitochondria appeared retained with a spheroidal morphology (denoted by red arrows), while those located in cytoplasm/lysosmes were markedly degraded (denoted by yellow arrows), which may be caused by different pH values of these organelles.

### *In vitro* cellular toxicity

In order to have a comprehensive evaluation of the biocompatibility of Mn@CaCO_3_/ICG@siRNA, the cytotoxicity of the nanoplatform on cancer cells was determined by CCK-8 assay and the effects on cell proliferation was observed by Real-Time Cell Analyzer. The CCK-8 results were shown in Figure 2D. After 24 h incubation with the lewis cells, the nanoplatform showed no significant cytotoxicity in the concentration range of 0-32 μg. Therefore, a dose of 16 μg/mL ICG was used for the rest of the study. Besides, similar results were obtained from the cell proliferation assay (Figure S10); the nanoplatform showed negligible adverse effect on the cell proliferation rates even at longer incubation times, without obvious difference on the proliferation curves compared with PBS group. These results demonstrate that the nanoplatform has an excellent biocompatibility making it suitable for further therapeutic applications.

In principle, when Mn@CaCO_3_/ICG@siRNA is exposed to the tumor microenvironment, the CaCO_3_ layer and MnO_2_ core immediately degrades. As a result of this degradation, O_2_/CO_2_ gas is produced. Moreover, this degradation would accelerate the ICG release and assist its resuspension into monomers, thereby facilitating a strong ICG penetration. Herein, 3D Multicellular tumor spheroid (MCTS) with Lewis cells were used in order to assess the delivery and penetration capacity of the Mn@CaCO_3_/ICG@siRNA 32. The fluorescence images of the horizontal section of the 3D MCTS are shown in Figure S11, a thin layer of ICG adhered to the 3D MCTS and penetrated into the 3D MCTS in the free ICG groups. However, for the nanoplatform groups, the penetration depth of ICG was significantly thicker than that of the free ICG, indicating that this approach significantly improved the delivery and penetration capacity of the loaded drug. The enhanced delivery and penetration capacity could be attributed to both the protection of the drug and the H^+^/H_2_O_2_ induced degradation properties of the nanoplatform.

### Western blotting

Based on the results of cell uptake, a western blotting (WB) experiment was performed to further verify the delivery ability of siRNA, and the concomitant PD-L1 silencing efficiency by Mn@CaCO_3_/ICG@siRNA delivered into Lewis cells. Before the western blot experiment, we analyzed the expression of the PD-L1 on lewis cells using flow cytometry. As shown in Figure S12, the ratio of PD-L1 expression reached 87.85%. The following WB results of Lewis cells after incubation with free siRNA and the nanoplatform for 24 h are shown in Figure 2E. The free siRNA does not show obvious effects on the protein expression of PD-L1. In contrast, the PD-L1 expression in the nanoplatform group gradually decreased as the amount of delivered siRNA increased, indicating a high silencing efficiency of the nanoplatform on the protein expression of PD-L1. And more, the ratio of PD-L1 expression dropped from 85.0% to 27.1% after siRNA PD-L1 transfection with the nanoplatform (Figure S13).

### * In vitro* photodynamic therapy

**Figure 3:**
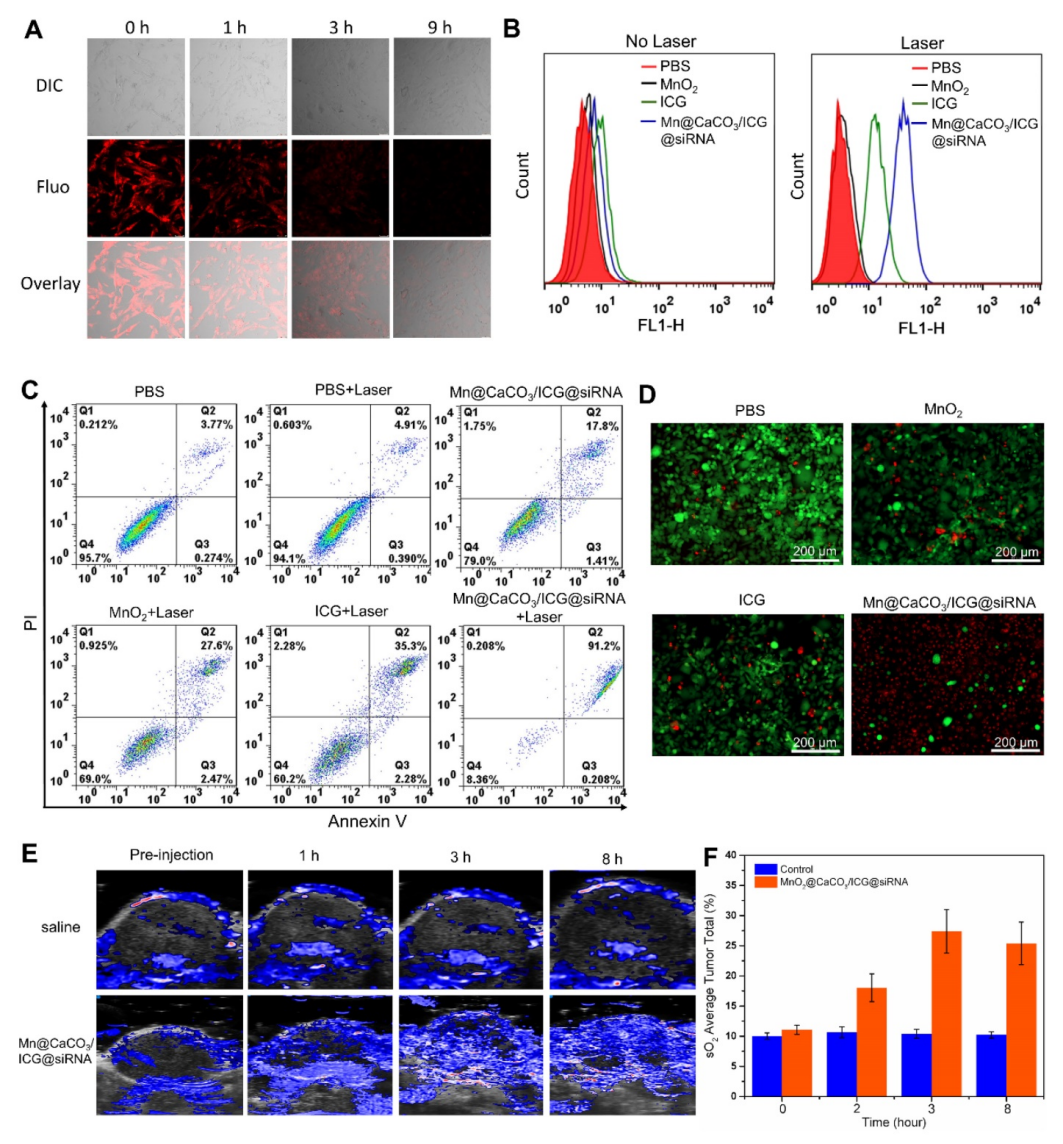
** (A)** Confocal fluorescence images showing increased intracellular O2 level after incubated with various concentration of MnO2@CaCO3/ICG for 12 h. **(B)** Flow cytometric analysis of LLC cells incubated with PBS, free ICG, MnO2 and MnO2@CaCO3/ICG@siRNA for 12 h, followed by 808 nm laser irradiation (0.5 w/cm2) for 3 min. **(C)** Flow cytometric analysis of LLC cells death with different treatments. **(D)** Fluorescence images of Calcein AM/PI stained LLC cells incubated with PBS, MnO2, free ICG and Mn@CaCO3/ICG@siRNA respectively for 12 h after the irradiation of 808 nm laser (6 min, 0.5 W/cm2). **(E)** *In vivo* photoacoustic imaging. The PA images of tumor regions of LLC tumor-bearing mice at different time-point after i.v. injection of saline or Mn@CaCO3/ICG@siRNA showing the oxygen saturation (sO2) signals. **(F)** Quantification of the sO2 signal intensity of the tumor region.

Considering that a portion of the internalized nanoplatform located inside the mitochondria and did not undergo H^+^/H_2_O_2_-mediated degradation, we further investigated whether tumor cells under the laser irradiation will produce ROS. O_2_ acts as the source for ROS production, and thereby the generation of O_2_ inside the cells was firstly detected using the oxygen-sensing probe [(Ru (dpp)_3_)]Cl_2_ after incubation with the nanoplatform for different periods of times. As shown in Figure 3A, the red fluorescence of [(Ru (dpp)_3_)]Cl_2_ gradually decreased as incubation time went by, and almost no red fluorescence was observed after 9 h, suggesting that the nanoplatform could significantly increase the amount of oxygen inside the cells. In the next experiment, ROS generated by Mn@CaCO_3_/ICG@siRNA in Lewis cells were monitored by DCF-DA. The results from flow cytometry (Figure 3B) revealed that the intracellular fluorescence intensity of Lewis cells treated with Mn@CaCO_3_/ICG@siRNA was almost 2.7-fold greater compared with free ICG, indicating that Mn@CaCO_3_/ICG@siRNA has a greater capacity to produce ^1^O_2_ than free ICG under our experimental conditions. ^1^O_2_ plays a crucial role in the efficacy of PDT. Therefore we can expect that the increased ^1^O_2_ production capacity of our nanoplatform would lead to a more efficient therapeutic effect.

Based on the good biocompatibility, efficient cellular uptake property and high ^1^O_2_ generating capacity of Mn@CaCO_3_/ICG@siRNA in Lewis cells, the nanoplatform was then applied for laser-mediated PDT * in vitro*. As expected by flow cytometry, it exhibited a distinct therapeutic effect on Lewis cells (Figure 3C). The minimum level of apoptosis and necrosis accounting for < 2 % were observed in the cells treated with PBS after laser treatment, indicating that the basal power density of laser was safe enough for the cell line. On the contrast, the apoptosis experimental results showed that Mn@CaCO_3_/ICG@siRNA produced better PDT effect with the cell mortality reaching up to 91 % than free ICG and MnO_2_ upon 808 nm laser irradiation. Consistent with the results of flow cytometry, the double-staining confocal images (Figure 3D) showed that almost no damaged cells were observed in groups of Lewis cells treated with PBS, ICG or MnO_2_. However, there were nearly equal amounts of dying cells (stained in red) and live cells (stained in green) in the ICG groups, indicating that free ICG had a partial effectivity for the killing of Lewis cells. The Mn@CaCO_3_/ICG@siRNA groups, however, showed that most of Lewis cells were not viable. Moreover, the enhanced photodynamic therapeutic effect of the nanoplatform was further verified* in vitro* by CCK-8 assay (Figure S14). The distinct difference in the capacity to produce ^1^O_2_ between Mn@CaCO_3_/ICG@siRNA and free ICG contributes to the enhanced therapeutic effect of Mn@CaCO_3_/ICG@siRNA mediated PDT * in vitro*.

### * In vivo* imaging

The effective accumulation of the nanoplatform and the enhancement of oxygen content in tumors are crucial for the nanoplatform-mediated enhanced PDT. In order to investigate whether the nanoplatform can effectively accumulate in tumors and improve the hypoxic microenvironment of the tumor tissue, we monitored in real-time the dynamic changes of oxygen content in the tumor site after injections of PBS and Mn@CaCO_3_/ICG@siRNA using Photoacoustic (PA) Imaging System under the oxygen measurement model 33. It can be seen in Figure 3e that the oxygen saturation of the tumor tissue is about 10 % O_2_ pre-injection in the two groups. However, the oxygen saturation signal in the group treated with our nanoplatform emerged 1 h post-injection, with the strongest PA signal at the peak of 36% appearing 6 h post-injection, and then gradually reduced to 35 % at 8^th^ h post-injection. Interestingly, the PA signal revealed that the oxygen was mainly generated within the tumor periphery in the first 1 h post-injection and was gradually generated at the tumor core site 3 h post-injection. As time went by, the hypoxic condition in the entire tumor site was significantly improved, as well as at the tumor periphery (Figure 3E and F). No obvious changes were detected in the saturation signal of oxygen for the tumor treated by saline during the whole monitoring process. These results proved that the nanoplatform can effectively accumulate in the tumor site through i.v. injection and then generate oxygen by reacting with H_2_O_2_ to produce extra O_2_ in and out the cancer cells, thereby significantly improving the hypoxic environment of the entire tumor tissue.

**Figure 4:**
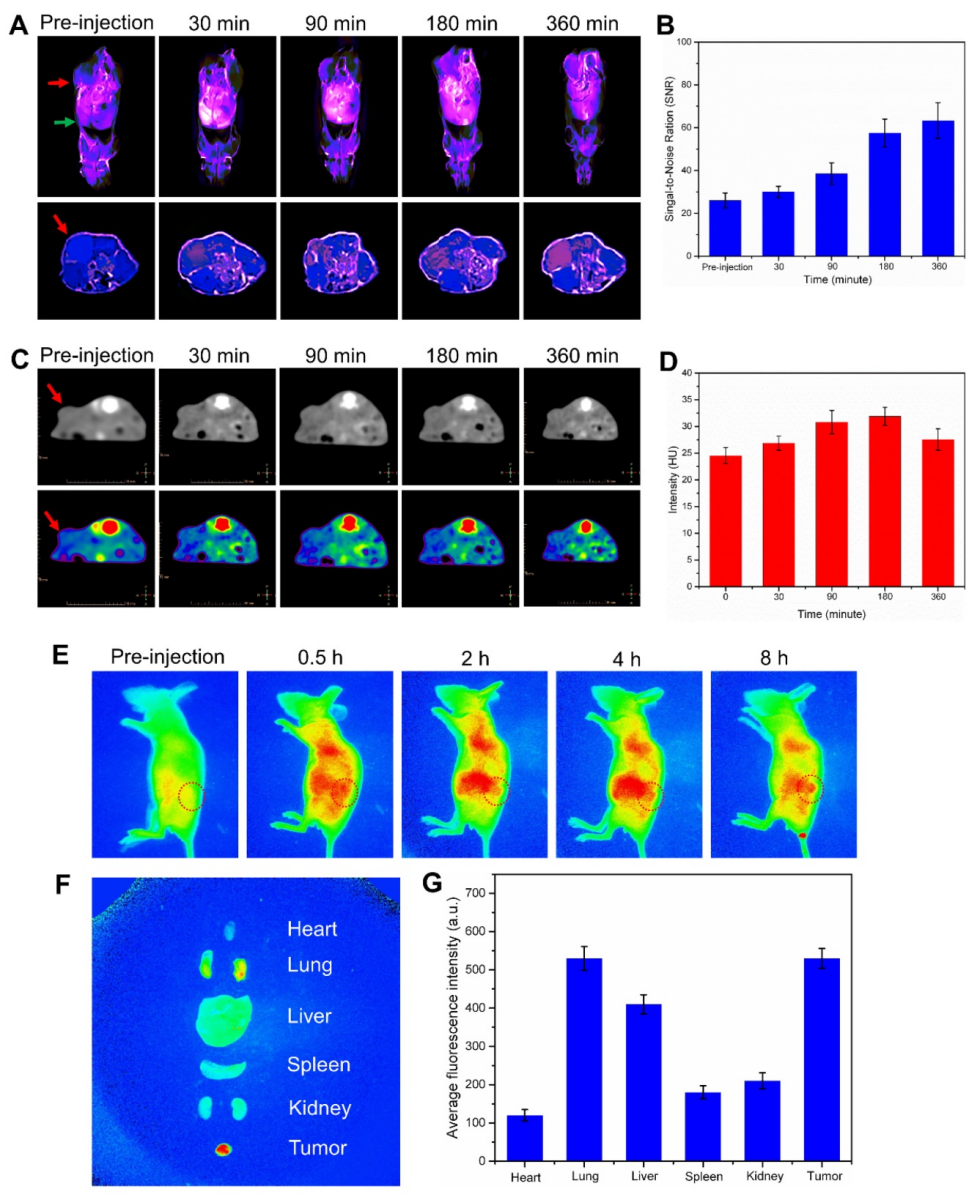
** Biodistribution and excretion of Mn@CaCO3/ICG@siRNA in LLC tumor-bearing mice. (A**) * In vivo* T1-weighted MR imaging of LLC tumor-bearing mice before and after intravenous administration of Mn@CaCO3/ICG@siRNA. Red arrows point the tumor region, and green arrow points the liver region. Upper images show the longitudinal section of the mice, while lower images show the transverse section. **(B)**. Quantitative analysis of the T1-MR signals from the tumor region. **(C)** Transverse CT images of LLC tumor-bearing mice. The arrows in the CT images indicate the tumor region. **(D)** Quantitative region of interest analysis (CT intensity) of tumor pre-injection and post-injection of Mn@CaCO3/ICG@siRNA. **(E)** Real-time fluorescence imaging of LLC tumor-bearing mice before and after i.v. injection of Mn@CaCO3/ICG@siRNA. Red circles indicate the region of the tumor. **(F)** Ex vivo fluorescence images of mice organs or tissue. **(G)** Quantitative analysis of the fluorescence signals.

Subsequently, based on the multimodal imaging abilities of the nanoplatforms, their time-dependent distribution, degradation and excretion with in Lewis tumor bearing mice, especially in the tumor and liver region, were observed through MR imaging /CT imaging /fluorescence imaging. When the nanoplatform reached the tumor site and the degradation kicked off by the H^+^/H_2_O_2_ environment, the degraded MnO_2_ can be used as T1 contrast agent for MR imaging 25. Therefore, we measured the T1 relaxation time of the nanoplatform and recorded T1-weighted MR images of the nanoplatform at different concentrations and media, as shown in Figure S15A and B. Next, the * in vivo* degradation and excretion of the nanoplatform were real-time monitored using a T1-MR imaging system. The T1-MR images are shown in Figure 4A and B, a strong T1-MR signal was observed in the liver 30 min post-injection, and this signal grew stronger as time went on. However, the T1-MR signal from the liver region gradually became weaker since 90 min after injection and only a faint signal was left at the last time-point, 360 min. On the other hand, the T1-MR signal from the intestinal region gradually became stronger after i.v. injection and reached a peak 360 min post-injection. The changes in T1-MR signals from liver and intestines revealed that the degraded nanoplatform was mainly expelled ed out from the organism via the hepato-biliary system (HBS)-feces pathway. The T1-MR imaging results proved that the nanoplatform can efficiently accumulate in the tumor site and that the degraded nanoplatform is able to be easily excreted from the body without being captured by the reticuloendothelial system (RES). And more, the blood circulation of nanoplatform was analyzed by measuring Mn content in blood by ICP-MS (Figure S16).

One of the components of the nanoplatform, calcium carbonate (CaCO_3_), a primary structural material for animal hard tissues such as bones and teeth, has a strong CT signal. Therefore, CT imaging is considered an outstanding technique to observe the accumulation, distribution and excretion of our CaCO_3_-based nanoplatform within the body. Thus, the * in vivo* CT imaging of the nanoplatform in the tumor tissue was conducted. CT images and signal results are shown in Figure 4 C and D. It can be seen that the CT signal from the tumor tissue was gradually increased and reached its maximum 180 min after i.v. injection. The signal began to weaken due to the degradation of CaCO_3_ induced by the acidic environment of the tumor, indicating that our nanoplatform had a tumor-responsive degradation characteristic.

Finally, the fluorescence distribution on Lewis tumor-bearing mice emitted by ICG were monitored within 8 h by i.v. injection with Mn@CaCO_3_/ICG@siRNA. The fluorescence images are shown in Figure 4E. After i.v. injection with Mn@CaCO_3_/ICG@siRNA, an evident fluorescence signal occurred in the liver and abdomen. Over time, the fluorescence signal from ICG in the liver became weakened gradually, and no obvious fluorescence signal could be observed 8 h post-injection, most likely because the released ICG could be excreted from the liver to the intestine as the nanoplatform degradation. A weak fluorescence signal was emerged in the tumor region after 1 hour injection, and then gradually increased as time went by, finally reaching a maximum level 8 h post-injection. Furthermore, in order to provide complementary insights about the distribution of ICG, an *ex vivo* fluorescence imaging of the nanoplatform was conducted in the main organs and the tumor tissue 8 h after i.v. injection. It could be clearly seen from Figure 4F and J that the nanoplatform efficiently delivered the loaded ICG to the tumor tissue. These fluorescence imaging results demonstrated that Mn@CaCO_3_/ICG@siRNA had a remarkable tumor-targeted delivery ability by promoting significantly the stability of the loaded-ICG molecules in the blood circulation and improving the selective and targeting accumulation in the tumor tissue.

### Immune responses

**Figure 5:**
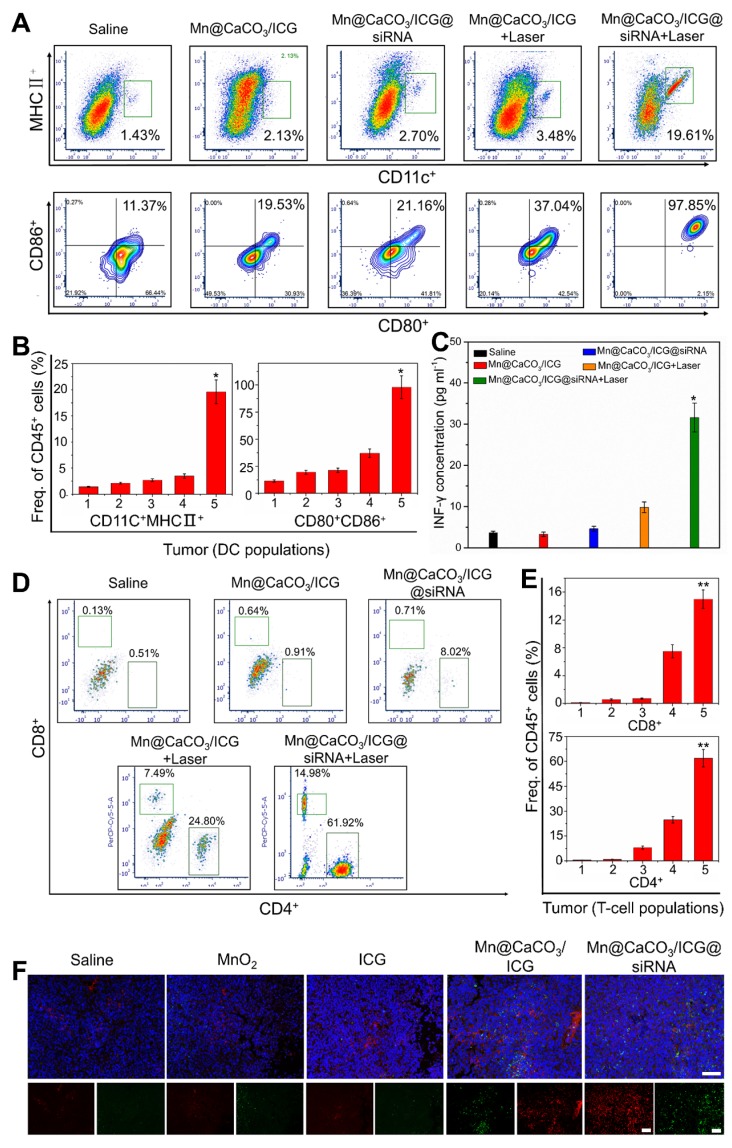
** Immunological responses after laser irradiation treatment with Mn@CaCO3/ICG@siRNA. (A)** and **(B)** Flow cytometric analysis of tumor-infiltrating DC cells in LLC tumor-bearing mice 4 days after laser irradiation. From 1 to 5: saline, Mn@CaCO3/ICG, Mn@CaCO3/ICG@siRNA, Mn@CaCO3/ICG + Laser and Mn@CaCO3/ICG@siRNA + Laser groups, respectively. **(C)** The levels of IFN-γ in each group (n=5 per group, mean ± s.d., ANOVA with Tukey's post-hoc test, *p < 0.01, **p < 0.001). **(D)** and **(E)** Representative flow cytometry data of tumor-infiltrating CD4+ vs CD8+ T cells in the LLC tumor-bearing mice 4 days after laser irradiation. From 1 to 5: saline, Mn@CaCO3/ICG, Mn@CaCO3/ICG@siRNA, Mn@CaCO3/ICG + Laser and Mn@CaCO3/ICG@siRNA + Laser groups, respectively. **(F)** Representative immunofluorescence images of tumor slides showing CD4+ (green) and CD8+ (red) T cells infiltrating the tumor tissues. Scale bars are 100 μm.

Tumor growth and invasion involve highly proliferative hypo-immunogenic cells that evade the surveillance and killing of the immune system 34. Therefore, one of the keys to inhibiting tumor is to activate the anti-tumor immune system to monitor and kill the tumor cells. Recently, various intriguingly immunological strategies have been explored to cure cancer by activating the immune system. It is well known that the PD-1/PD-L1 signaling pathway leads to adaptive immune resistance. Herein, we attempted to block the immune resistance pathway by loading PD-L1 siRNA on the nanoprobes to down-regulate the expression of PD-L1 in tumor cells. To verify whether our treatment strategy could activate a tumoral immune response and inhibit tumor immune resistance, we detected the tumor immune responses induced by the nanoprobes after laser treatment using flow cytometry. Five days after i.v. injection, we firstly analyzed the number and maturity of dendritic cells (DC) which shows the strongest antigen presentation ability as antigen presenting cell (APC) infiltrating within tumor tissues and surrounding lymph nodes. As shown in figure 5A and B, tumors of those mice injected with Mn@CaCO_3_/ICG@siRNA showed significantly enhanced DC infiltration after laser irradiation, the occupancy ratio of which reached 19.61% changing from 1.43% when compared with that of the saline group. More importantly, 97.85% of these DC infiltrated within the tumor in Mn@CaCO_3_/ICG@siRNA group were in their mature forms, indicating that the therapeutic strategy could strongly stimulate the recruitment and maturation of DC cells after laser irradiation. Similar results were observed by analyzing DC obtained from lymph nodes surrounding the tumor (Figure S17 and S18), the degree of infiltration (2.67%) and maturity (35.85%) of DC in the irradiated group treated with Mn@CaCO_3_/ICG@siRNA was the highest in each group.

Subsequently, the changes in secretion of IL-12 and IL-18 in serum of each treatment group was quantified by ELISA assay (Figure S19 and 20). The secretion of IL-12 and IL-18 in serum were significantly increased. The quantity of IL-12 in the nanoplatform-treated group was about 3.7 times bigger than that in saline group, while IL-18 after administration with the nanoplatform even increased by 3.1 times compared with the control group. These results indicated that DCs in mice after treated with the developed nanoplatform became matured and secreted a large amount of cytokines to recruit further immune response. Especially, the production of cytoxic T leukocyte (CTL) will consequently be induced to dominate the TH1-type immune response leading to the final killing and clearance of tumor cells. Thereby, we next analyzed T-cells infiltrating within the tumor tissues of LLC-tumor bearing mice. The flow cytometry analysis and statistical results are shown in Figure 5D and E. Both Mn@CaCO_3_/ICG and Mn@CaCO_3_/ICG@siRNA plus laser-irradiated mice showed a significant increase in effector/memory helper CD4^+^ T-helper cells and effector/memory cytotoxic CD8^+^ T-cytoxic cells in the tumor tissues 5 days after i.v. injection. And more, the immunofluorescence imaging further confirmed the accumulation of CD4+ and CD8+ T cells in the tumors from Mn@CaCO_3_/ICG@siRNA treated groups (Figure 5F). As shown in Figure S21, as expected, the proportion of T reg cells in the tumor tissue of the drug treatment group was the lowest in all treatment groups. The tumor infiltrating CD8^+^ cells directly kill tumor cells, while also enhance cytokines secreting. Therefore, we evaluated the tumor immune response-related cytokines- TNF-γ secreted by tumor- infiltrating CD8^+^ T cells. As expected, the highest expression of INF-γ was detected in the Mn@CaCO_3_/ICG@siRNA irradiated groups (Figure 5C), which further proved CTL-mediated tumor immunity induced by our strategy. Taken together, these results have fully demonstrated that the laser-mediated treatment with Mn@CaCO_3_/ICG@siRNA successfully activates CTL-mediated anti-tumor immunity for efficient immunotherapy in addition to directly killing tumor cells by PDT.

### *In vivo* therapy

Encouraged by the capability of the nanoplatform to efficiently improve the tumor′s hypoxic environment and activate tumor immune responses, we next investigated the * in vivo* antitumor therapeutic efficacy of the nanoplatform under laser irradiation. We first observed the pathological changes in the main organs of mice treated by the nanoplatform without laser irradiation. As shown in Figure S22, 7 days after i.v. injection, no obvious inflammatory lesion or damage were observed in main organs of the Mn@CaCO_3_/ICG@siRNA group, indicating that the nanoplatform exhibited few acute side effects and showed excellent biocompatibility. Meanwhile, the significant down-regulation for expression of PD-L1 protein was checked when compared with the saline groups (Figure S23).

**Figure 6:**
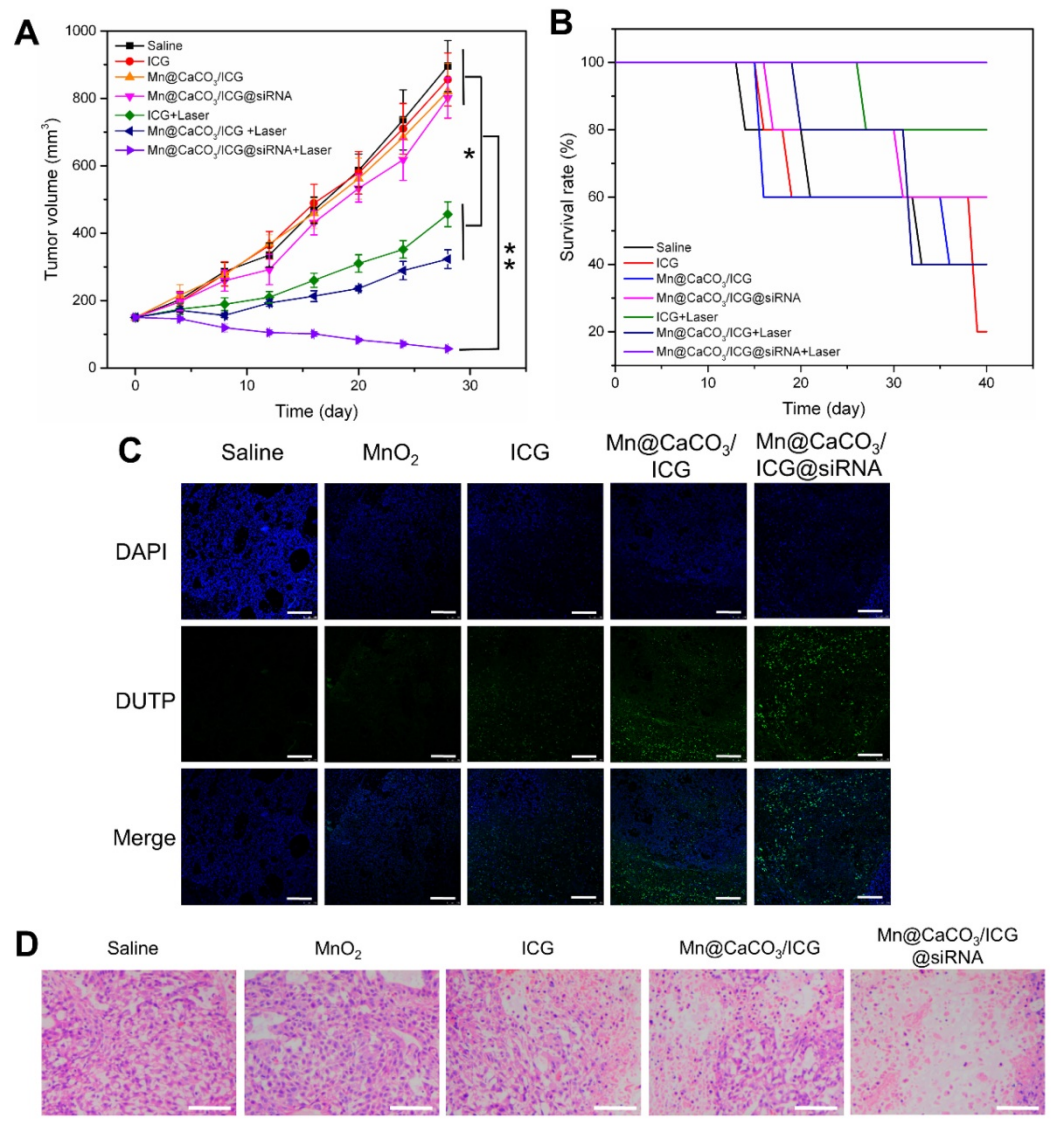
*** In vivo* laser-mediated therapy. (A)** Tumor volume in various groups after 808 nm laser irradiation. **(B)** Survival rate in various groups after laser treatment. **(C)** TUNEL assay (TdT-mediated DUTP nick end labeling). TUNEL stained tumor slices from each groups obtained 3 day after laser treatment. All scale bars are 100 μm. **(D)** Representative H&E sections of the tumor from each group collected after laser irradiation for 21 days. All scale bars are 200μm.

After the combined exertion of PDT and immunological therapy based on our nanoplatform, the treated mice were estimated by various experimental methods to evaluate the effect of such combined therapy strategy. As shown in Figure 6A, during the 20 days after the final laser treatment, there was an obvious rapid tumor volume growth in the saline treated group, and the survival rate up to 40% was the lowest among all groups (Figure 6B), suggesting that single laser irradiation was invalid to inhibit tumor growth. In accordance with the * in vitro* therapeutic results, either MnO_2_ or siRNA -based administration was proved to be fairly ineffective in inhibiting tumors * in vivo*. For the ICG injected groups, although the tumor growth showed a significant decrease and the survival rate also increased to a certain extent, ICG alone did not achieve the desired therapeutic effect. In contrast, the nanoplatform injected group showed the best effect on inhibiting the tumor growth and increasing the survival rate. To determine the deterioration of tumors cells after the coordinated enhanced PDT and tumor immunity killing, we conducted the TUNEL experiment. The results are shown in Figure 6C. A small amount of cancer cells stained in green could be observed in the ICG group. Much more dead cells could be observed in Mn@CaCO_3_/ICG group concomitant with a large number of living cells stained in blue, while great amounts of tumor cells in the Mn@CaCO_3_/ICG@siRNA group were killed after the laser-mediated treatment, however, hardly any no dead cell was observed in the saline and MnO_2_ group. In addition, H&E staining of tumor tissues sections collected from the treated mice after 20-day treatment were performed to obtain pathological changes (Figure 6D). Consistent with TUNEL results, no obvious damaged tumor cells occurred in the saline, MnO_2_ injected groups, while a minority of damaged tumor cells was observed in the group injected with ICG. It should be noted that a massive amount of tumor cells were destroyed in the Mn@CaCO_3_/ICG@siRNA injected group. These results fully demonstrate that the nanoplatform exhibits a strong synergistic effect of the enhanced PDT and anti-tumor immunity responses in inhibiting the tumor cells growth under laser irradiation.

## Conclusions

In summary, we have designed and fabricated a multifunctional theranostic nanoplatform using MnO_2_/CaCO_3_ nanoparticles loaded with PD-L1 siRNA characteristic of pH-induced degradation and oxygen generation for laser-mediated photodynamic immunotherapy in combination with the supportive immunotherapy. Benefitting from the ability of the nanoplatform to generate oxygen in the tumor microenvironment, and also thanks to the effective protection of the loaded cargo, an enhanced photodynamic therapeutic effect was obtained in both * in vitro* and * in vivo* experiments. This, in turn, further facilitated the activation of the anti-tumor immunity. Meanwhile, blocking PD-1/PD-L1 mediated immune evasion pathway by siRNA loaded on the nanoplatform activated the tumor immunity system and enhanced its anti-tumor efficiency. Overall, our nanoplatform has achieved a satisfactory photodynamic-immuno combined therapy to cure tumor. The nanoplatform addresses the critical problems of poor therapeutic effects of conventional PDT and tumor immune resistance due to tumor environmental characteristics, and offers a new strategy for combined PDT-immunotherapy to achieve effective treatments of malignant tumors.
