# Supplementary material for: A tumor microenvironment responsive biodegradable CaCO3/MnO2- based nanoplatform for the enhanced photodynamic therapy and improved PD-L1 immunotherapy
Source: Theranostics. 2019 Sep 21;9(23):6867–84. doi: 10.7150/thno.37586 (PMC6815945; doi:10.7150/thno.37586)
Supplement: Supplementary file 1 — Supplementary figures and tables. [file thnov09p6867s1.pdf]

A tumor microenvironment responsive biodegradable $CaCO_3/MnO_2$-based nanoplatform for the enhanced photodynamic therapy and improved PD-L1 immunotherapy

Yanlei Liu[1,2], Yunxiang Pan[1], Wen Cao[1], Fangfang Xia[1], Bin Liu[1], Jiaqi Niu[1], Gabriel Alfranca[1], Xiyang Sun[3], Lijun Ma[3], Jesus Martinez de la Fuente[1], Jie Song[1], Jian Ni[1], Daxiang Cui[1,2]*

[1]Institute of Nano Biomedicine and Engineering, Shanghai Engineering Research Centre for Intelligent Diagnosis and Treatment Instrument, Department of Instrument Science and Engineering, School of Electronic Information and Electrical Engineering, Shanghai Jiao Tong University, 800 Dongchuan Road, Shanghai 200240, P.R. China
[2]National Center for Translational Medicine, Collaborative Innovational Center for System Biology, Shanghai Jiao Tong University, Shanghai 200240, P. R. China
[3]Tongren Hospital, Shanghai Jiao Tong University School of Medicine, 1111 XianXia Road, Shanghai, 200336, China.
E-mail: dxcui@sjtu.edu.cn

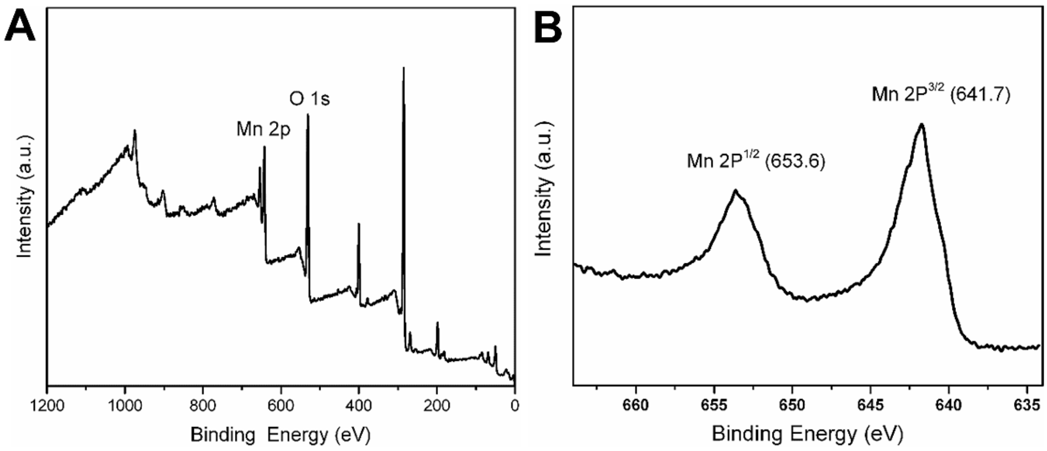

Supporting Figure S1. XPS analysis: (a) XPS spectra of the as-prepared MnO_2 nanoparticles. (b) Showing the high resolution Mn(2p) XPS spectra of Mn^{4+} in MnO_2.

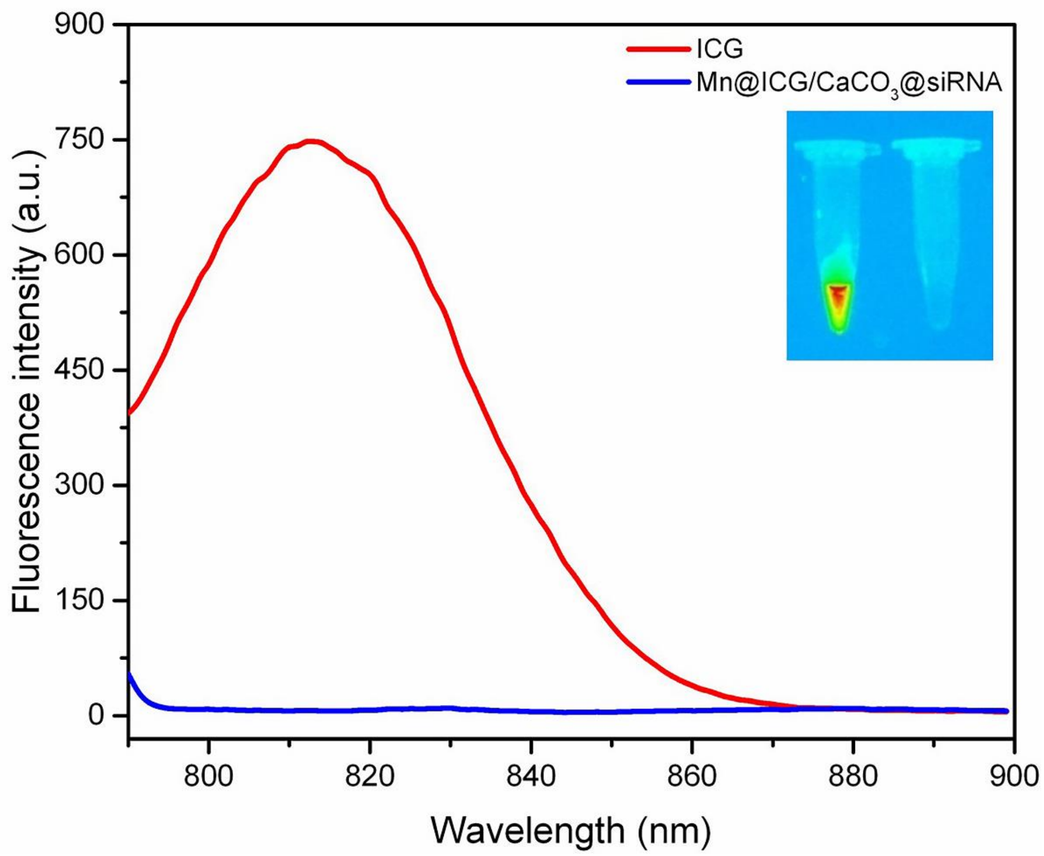

Supporting Figure S2. Fluorescence emission spectra of Mn@$CaCO_3$/ICG@siRNA at 710 nm excitation. Inset: fluorescence images of Mn@$CaCO_3$/ICG@siRNA (excitation=710 nm).

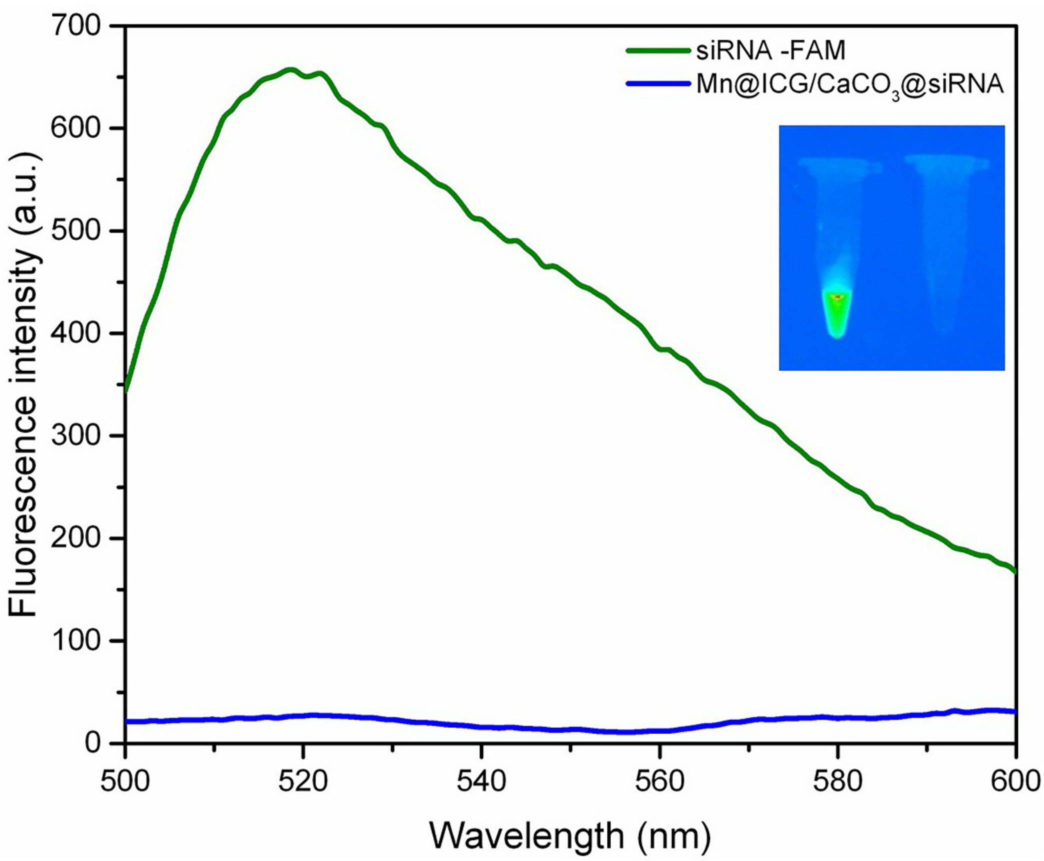

Supporting Figure S3. Fluorescence emission spectra of Mn@$CaCO_3$/ICG@siRNA at 375 nm excitation. Inset: fluorescence images of Mn@$CaCO_3$/ICG@siRNA (excitation=375 nm).

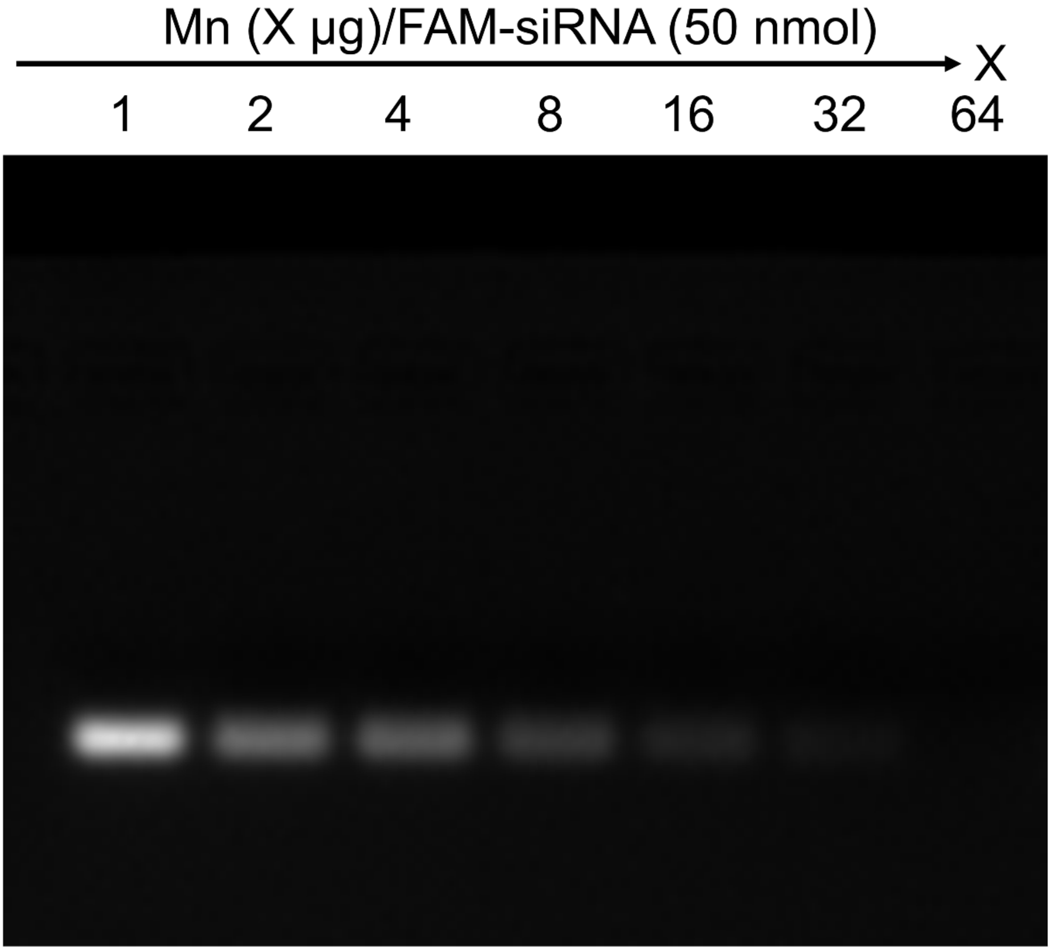

Supporting Figure S4. Polyacrylamide gel electrophoresis assay for the optimunim binding ratio of Mn@$CaCO_3$/ICG: FAM-siRNA. Mn@$CaCO_3$/ICG and free FAM-siRNA were mixed at the different Mn^{4+} /siRNA ratio, and analysed by polyacrylamide gel electrophoresis.

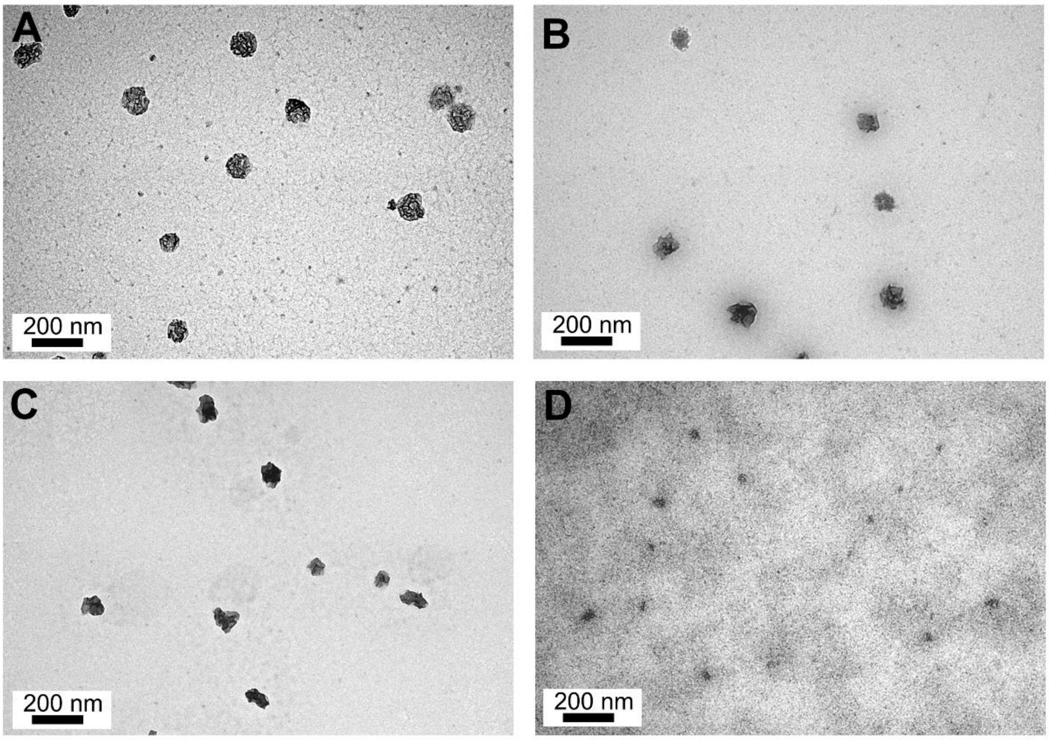

Supporting Figure S5. TEM images of Mn@$CaCO_3$/ICG@siRNA after incubation in DI water containing 50 μM H_2O_2 at pH values of 6.5 for various periods of time (0, 1, 3, 5 h).

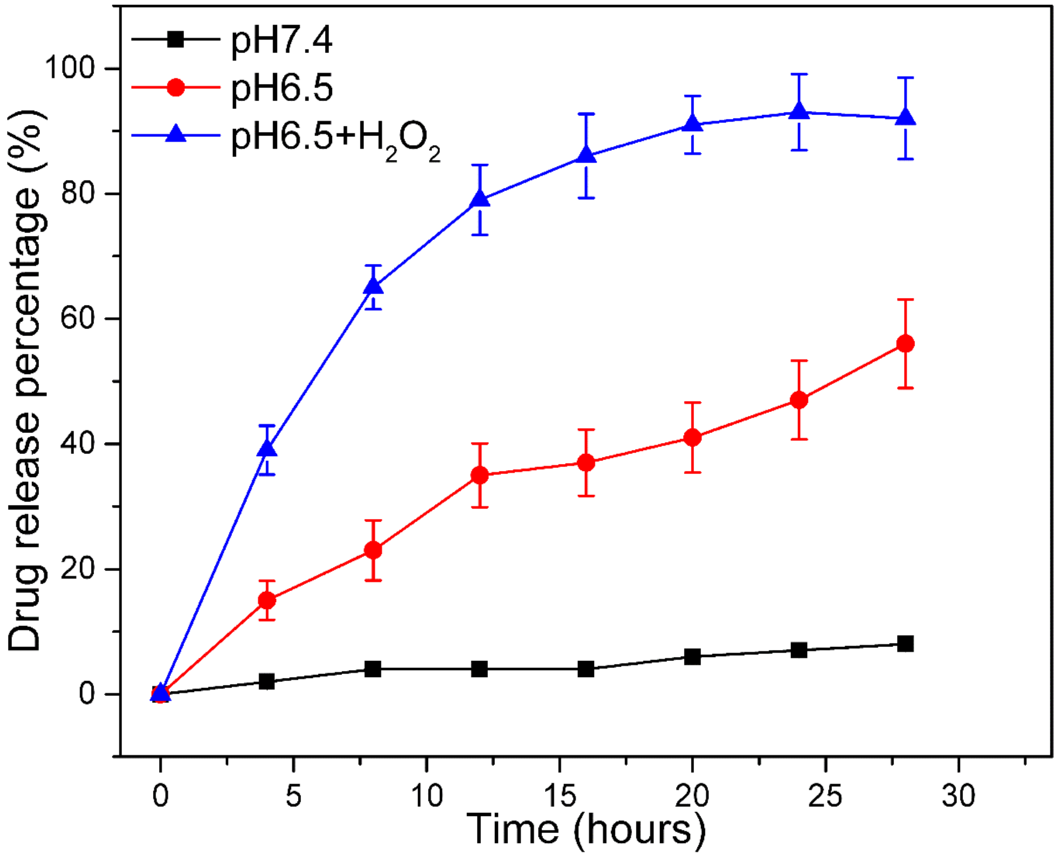

Supporting Figure S6. The release profiles of the calcium ions from Mn@$CaCO_3$/ICG@siRNA in the presence or absence of 50 μM H_2O_2 with different pH value (6.5, 7.4).

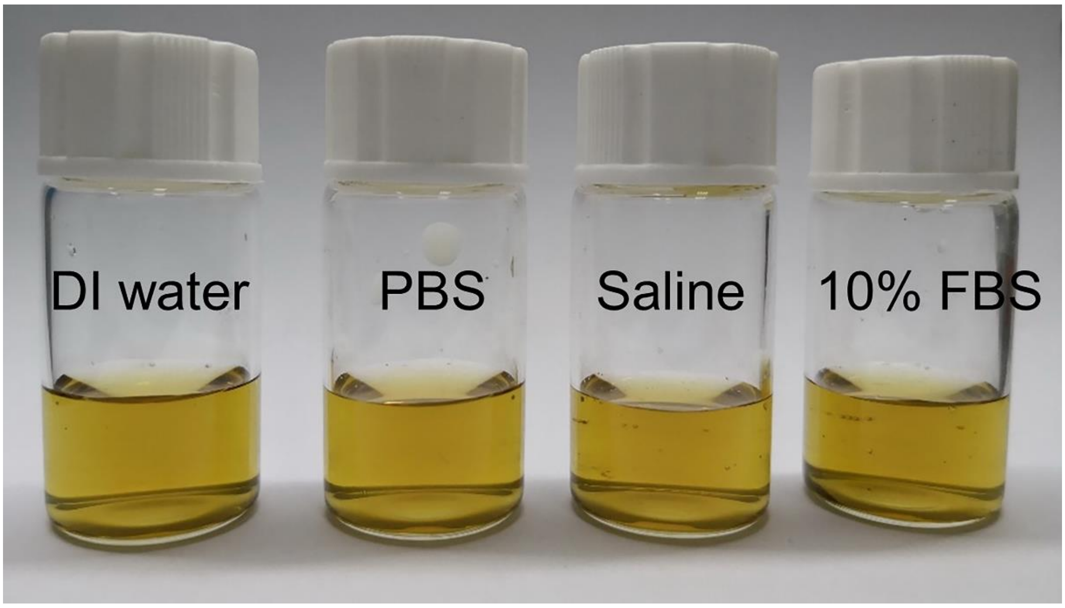

Supporting Figure S7. The digital photos of Mn@$CaCO_3$/ICG@siRNA dispersed in various aqueous media (from left to right: DI water, PBS, saline and saline containing 10% FBS) for 15 day.

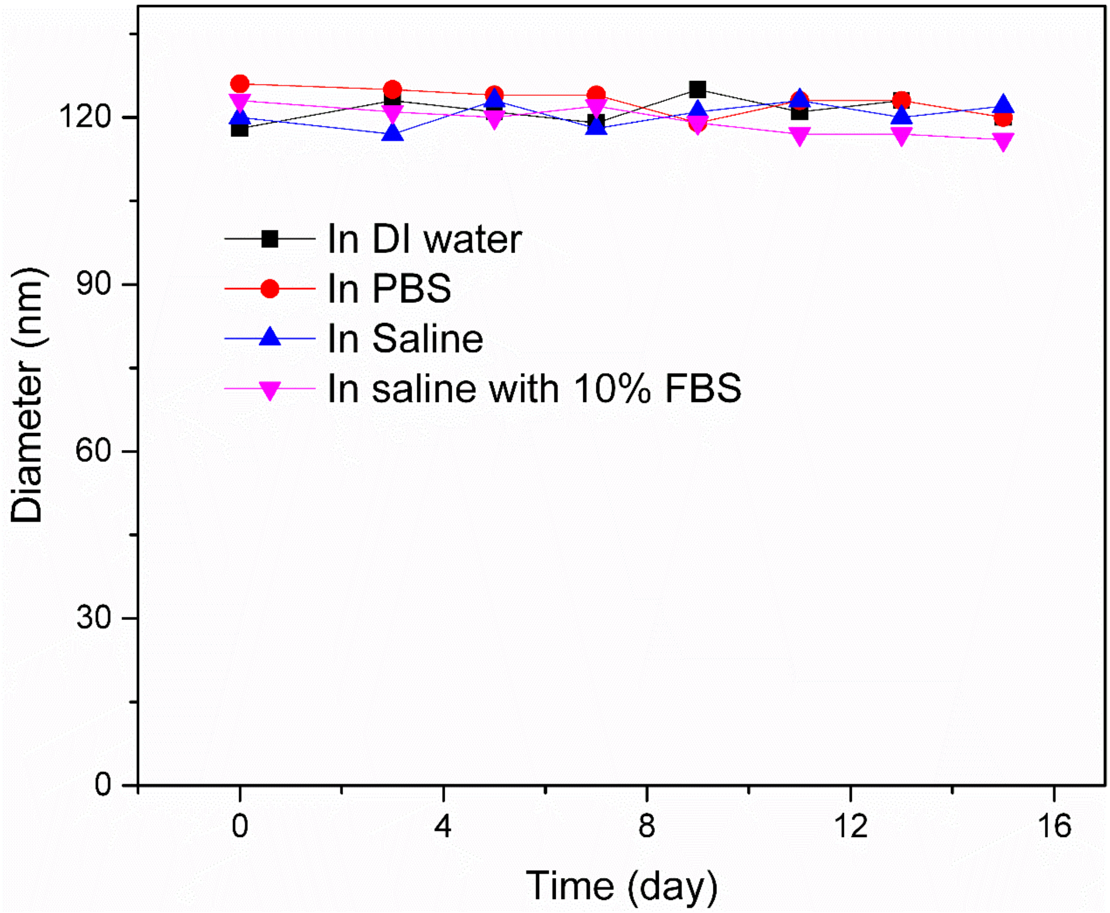

Supporting Figure S8. The size stability study of Mn@$CaCO_3$/ICG@siRNA with time in saline or DMEM containing 10% FBS. The DLS result showed that no obvious size changes of the nanoprobes incubation in saline or DMEM with 10% FBS for 15 day, indicating the nanoprobes are stable in these media.

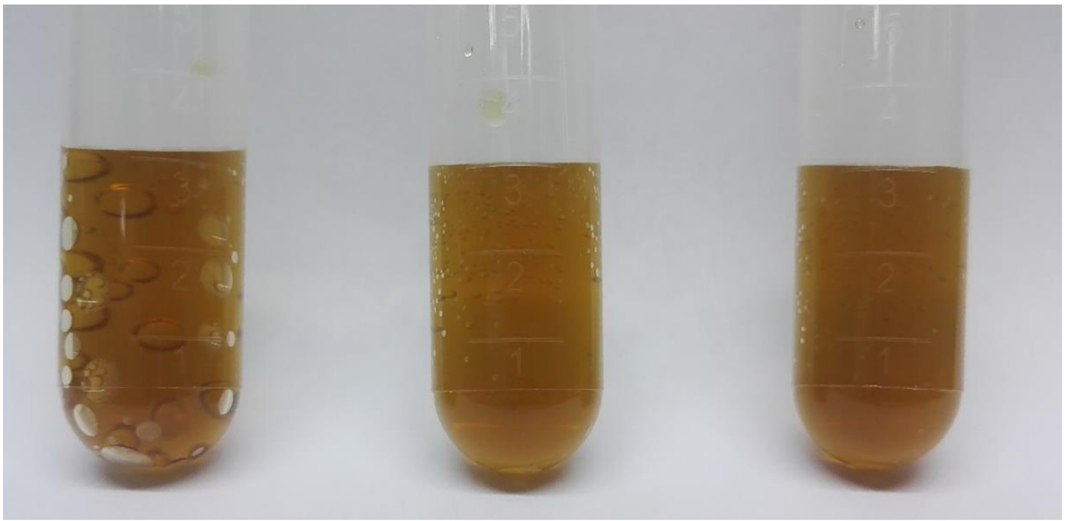

Supporting Figure S9. Digital photo of Mn@$CaCO_3$/ICG@siRNA incubated with H_2O_2 plus HCl, HCl and PBS for 2 min, respectively.

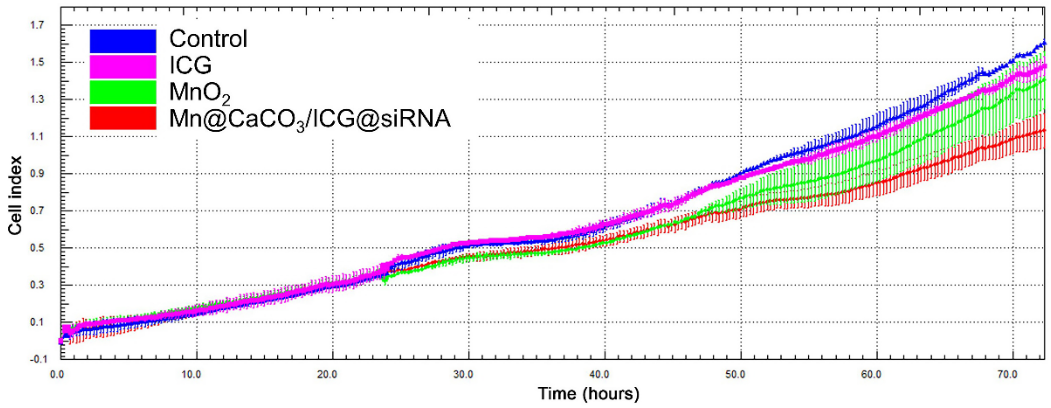

Supporting Figure S10. The effects of free ICG, MnO_2 and Mn@$CaCO_3$/ICG@siRNA on LLC cells.

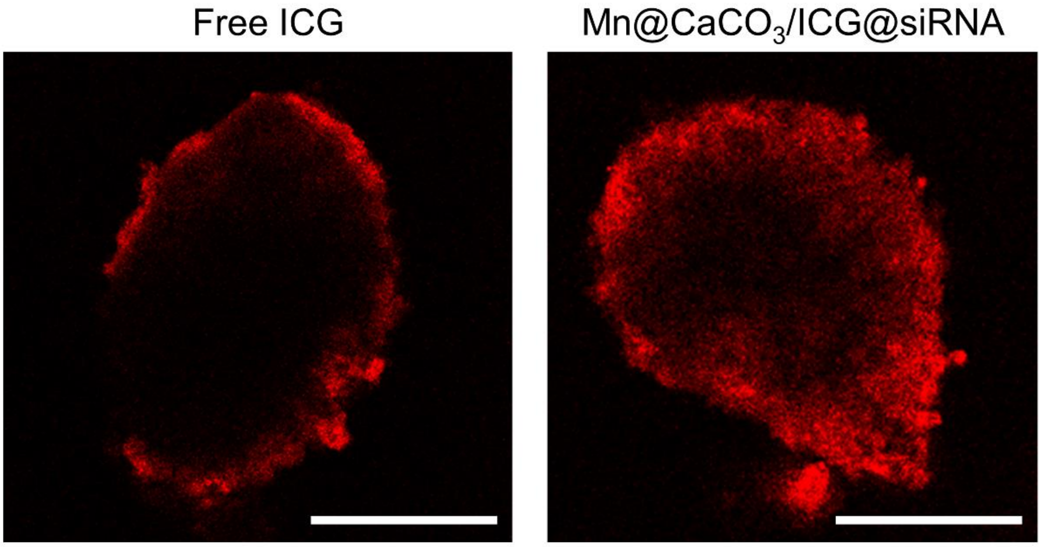

Supporting Figure S11. Penetration depth of free ICG and Mn@$CaCO_3$/ICG@siRNA in the 3D multicellular tumor spheroid (MCTS) model of lewis cells. Scale bars are 200 μm.

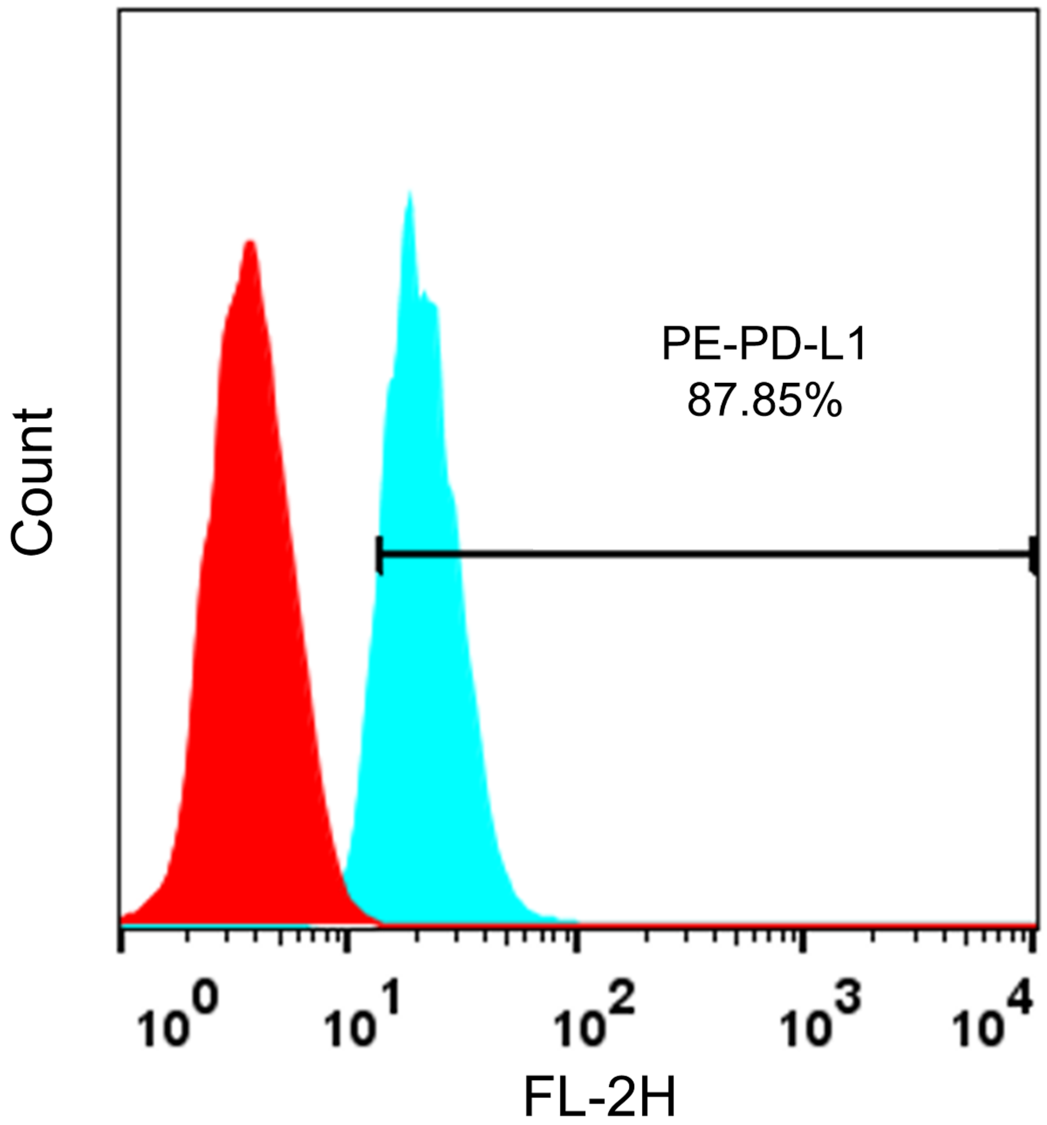

Supporting Figure S12. The expression of PD-L1 on the surface of Lewis cells.

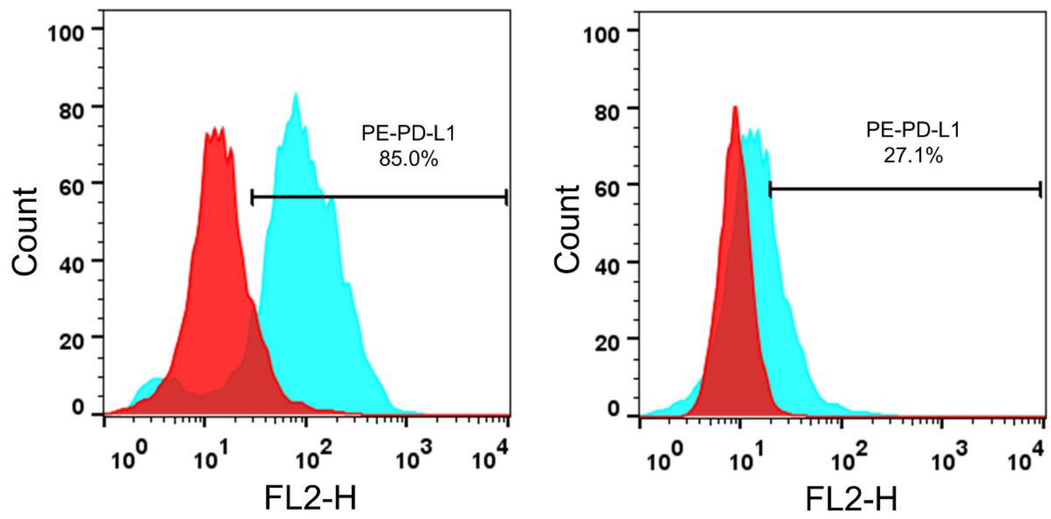

Supporting Figure S13. The expression of PD-L1 on the surface of Lewis cells before and after siRNA transfection by with nanoplatform (left: before, right: after).

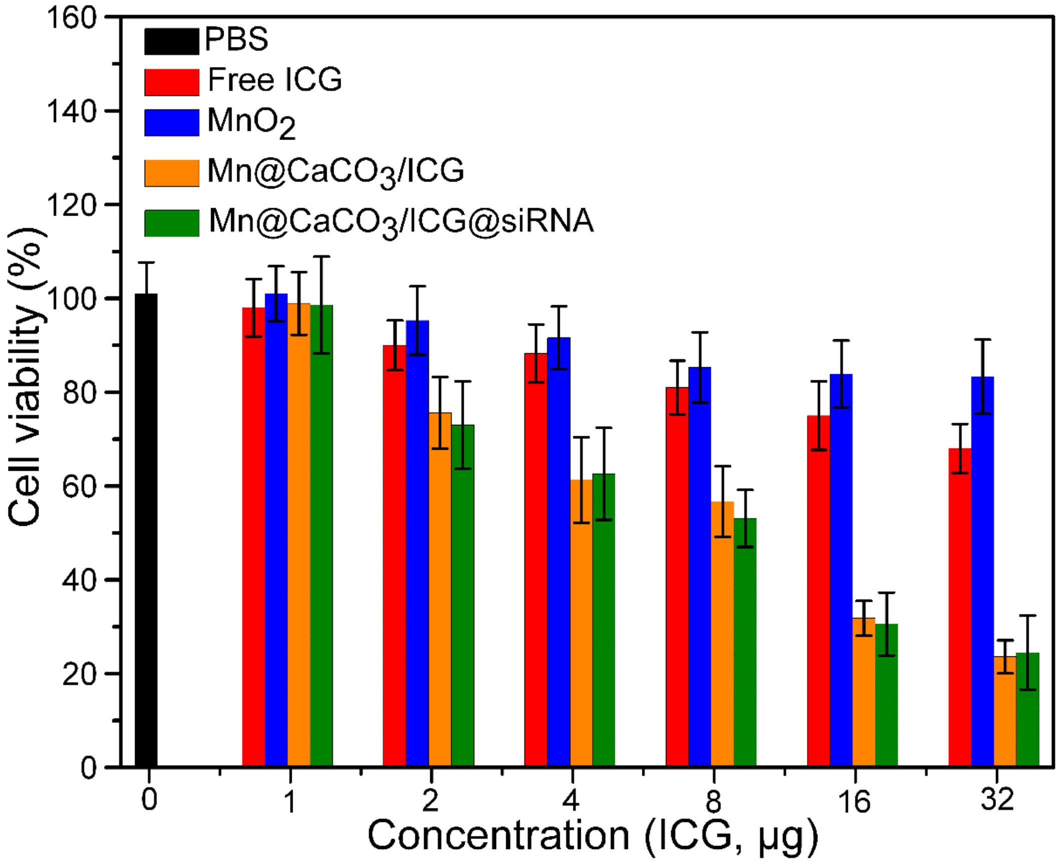

Supporting Figure S14. The cell viability of LLC cells incubated with with PBS, MnO_2, free ICG and Mn@$CaCO_3$/ICG@siRNA respectively for 12 h after the irradiation of 808 nm laser (6 min, 0.8 W/cm^2), which was determined by CCK-8 assay.

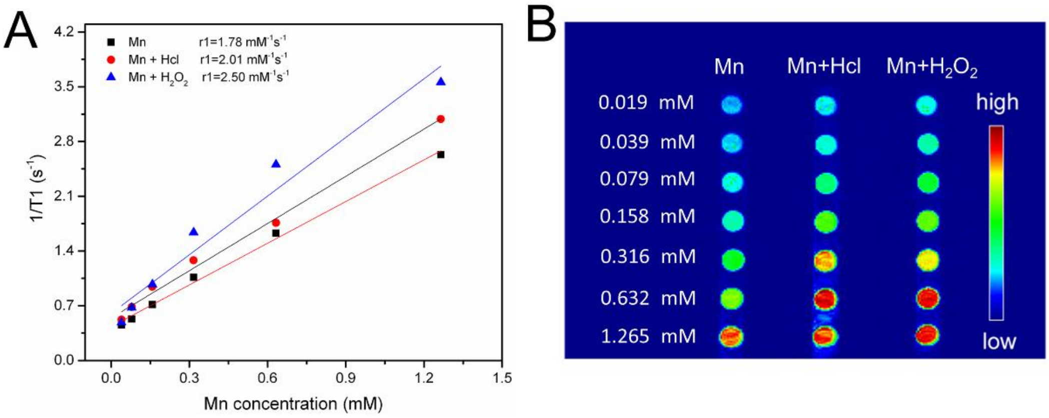

Supporting Figure S15. *In vitro* T1-weighted MR imaging. (a) The linear fitting of the inverse T1 of Mn@$CaCO_3$/ICG@siRNA after 12 h incubation in different buffer solution at a series of Mn^{4+} concentration. (b) The T1-MR images of Mn@$CaCO_3$/ICG@siRNA.

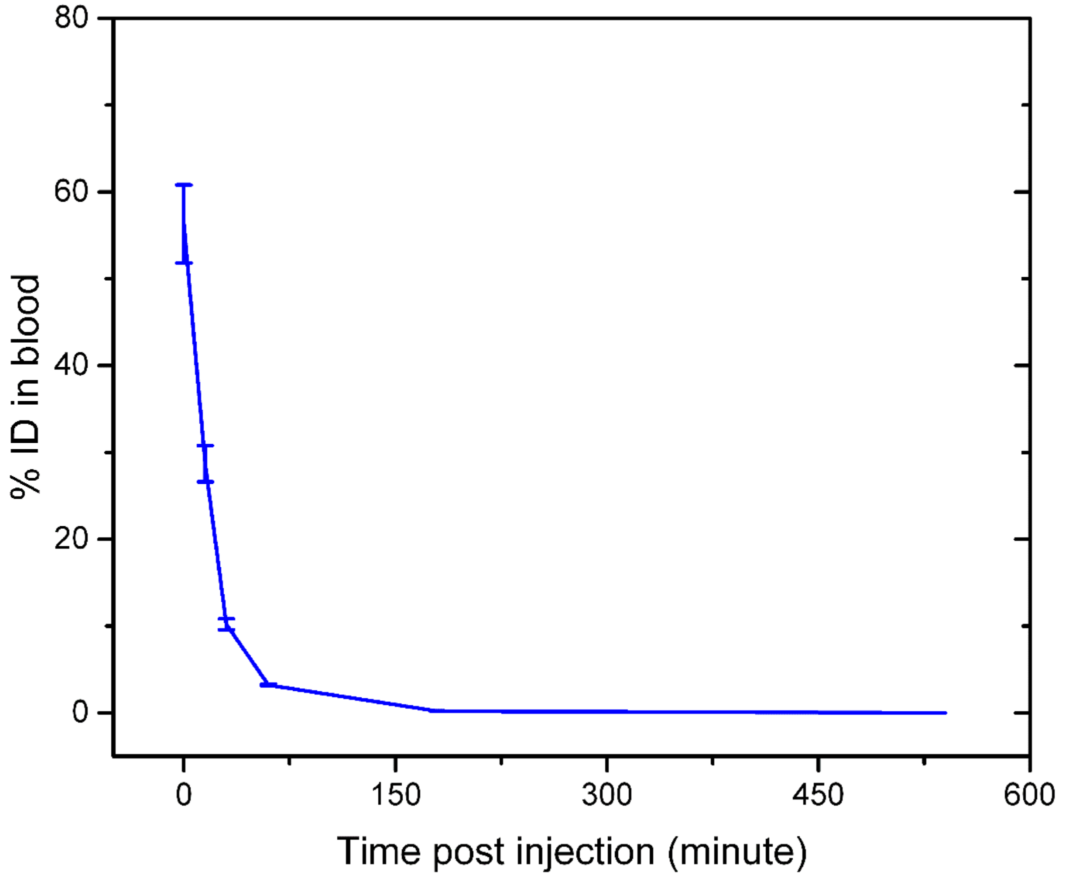

Suopporting Figure S16. The blood circulation of nanoplatform analyzed by measuring Mn content by ICP-MS.

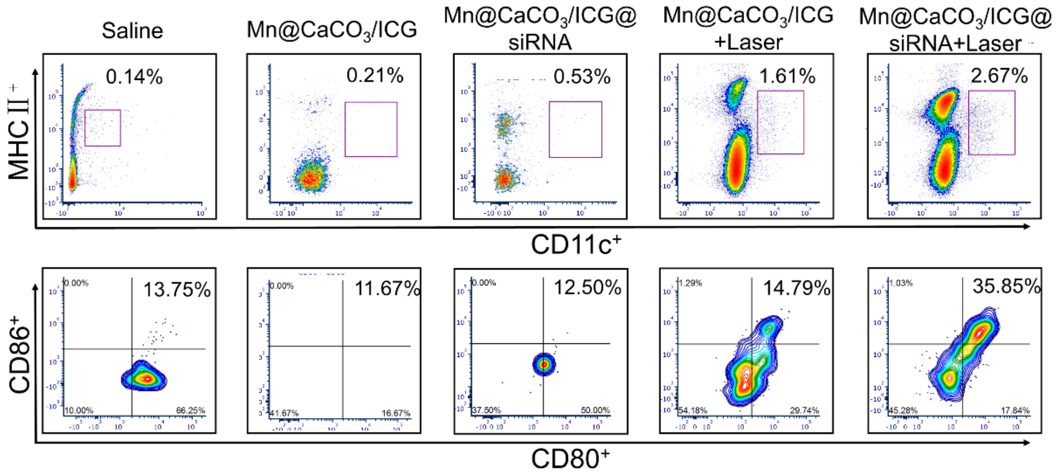

Supporting Figure S17. Flow cytometric analysis of DC cells drained from lymph nodes surrounding the tumor in the LLC tumor-bearing mice 4 days after laser irradiation.

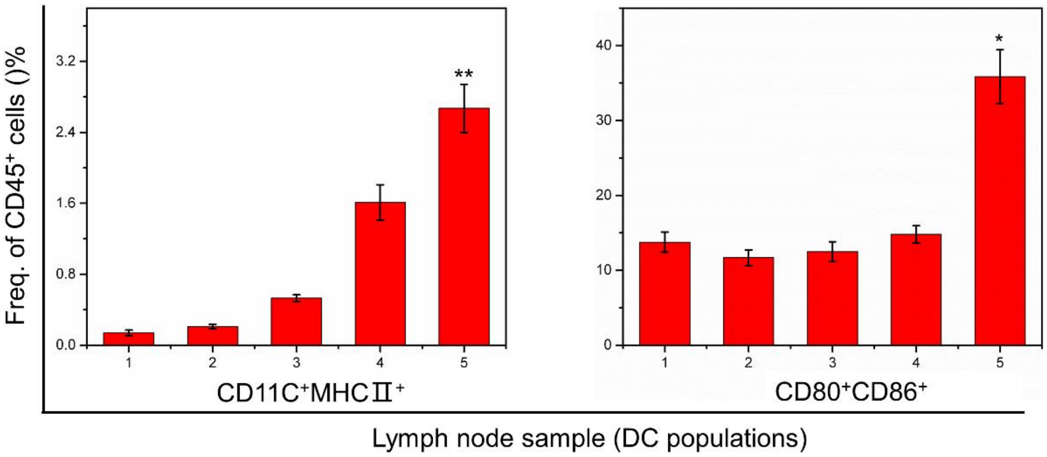

Supporting Figure S18. Representative flow cytometry date of DC cells drained from lymph nodes. n=5 per group, mean±s.d., ANOVA with Tukey's post-test, *$p < 0.05$, **$p < 0.001$.

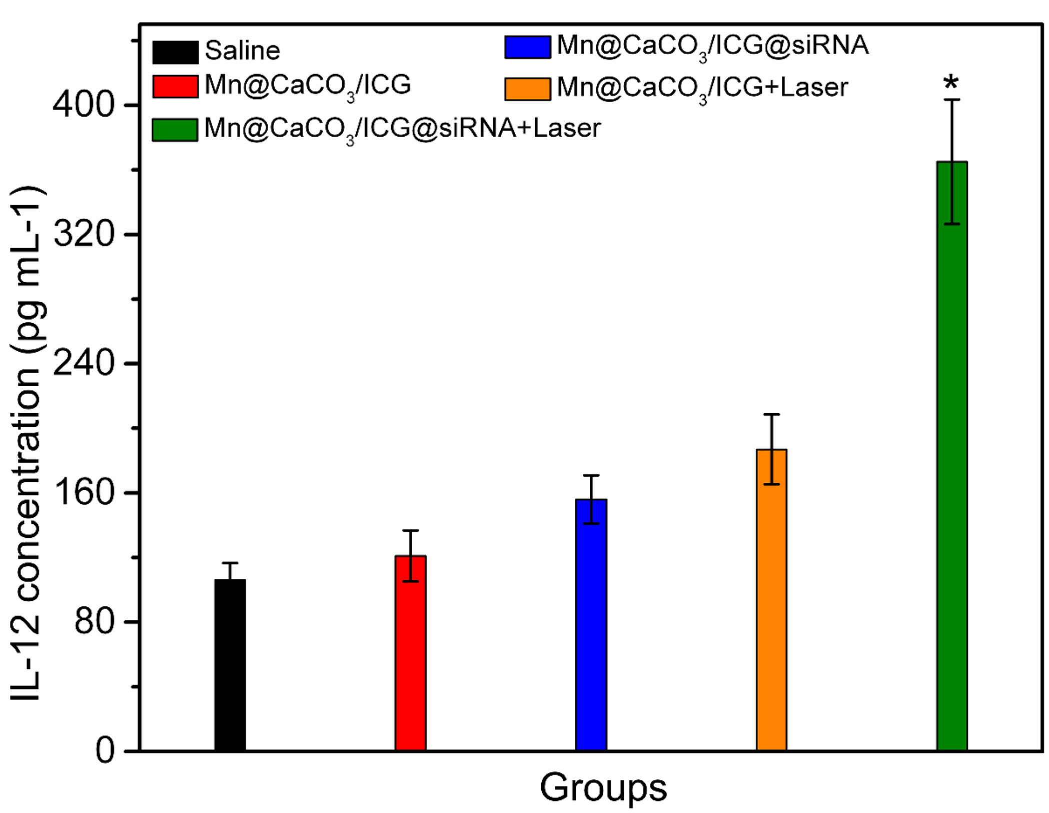

Supporting Figure S19. The level of IL-12 in the groups post various treatments. (n=5 per group, mean±s.d., ANOVA with Tukey's post-test, *p < 0.01, **p < 0.001). Compared with the saline group.

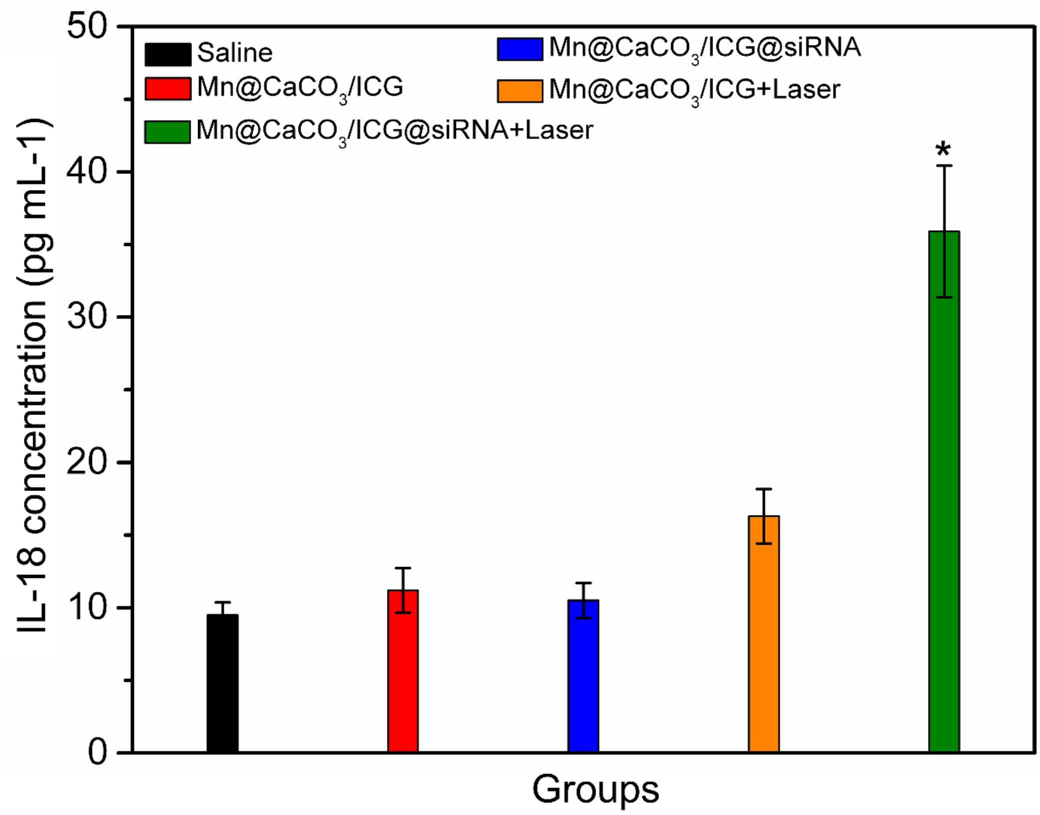

Supporting Figure S20. The level of IL-18 in the groups post various treatments. (n=5 per group, mean±s.d., ANOVA with Tukey's post-test, *p < 0.01, **p < 0.001). Compared with the saline group.

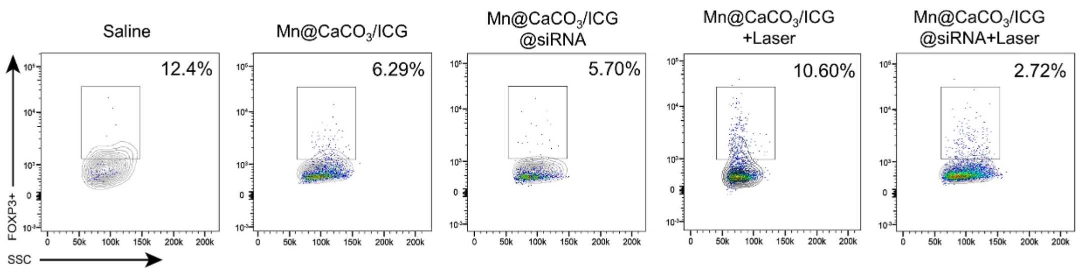

Supporting Figure S21. Flow cytometric analysis of T-reg cells drained from the tumor tissue in the LLC tumor-bearing mice 4 days after laser irradiation.

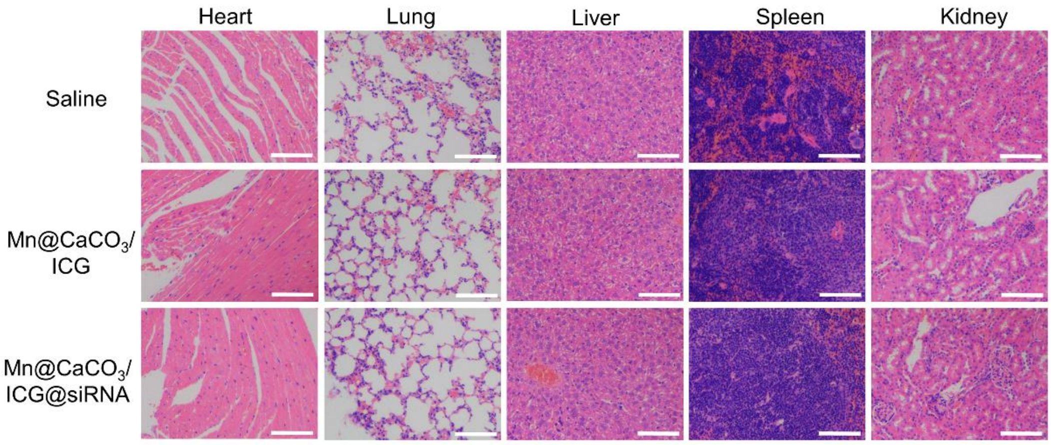

Supporting Figure S22. H&E stained organs (heart, liver, lung, spleen and kidney) slices from each groups collected 14 day after i.v injection of saline, Mn@$CaCO_3$/ICG and Mn@$CaCO_3$/ICG@siRNA. All scale bars are 100 μm.

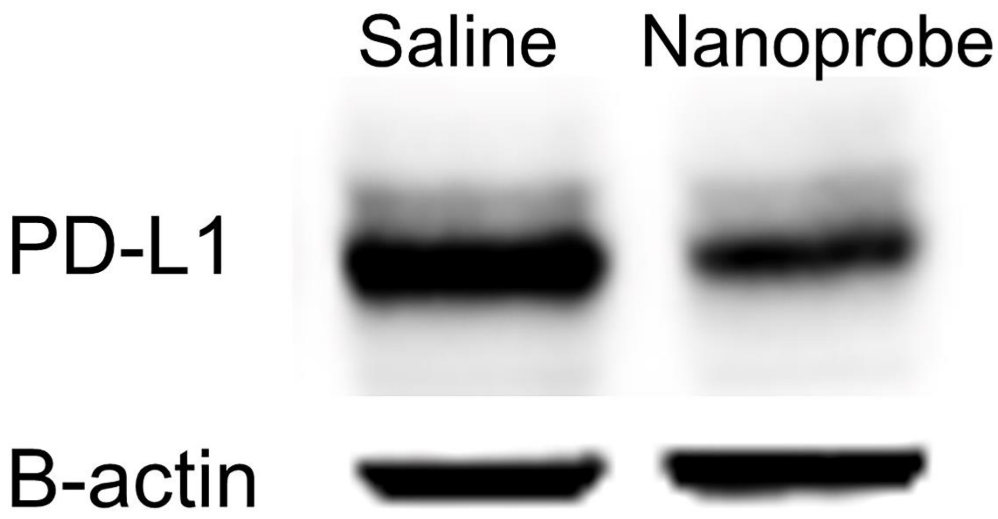

Supporting Figure S23. Western blot of PD-L1. Total protein harvested from normal tumor tissue was used as control.
